# Biostimulants in Plant Science: A Global Perspective

**DOI:** 10.3389/fpls.2016.02049

**Published:** 2017-01-26

**Authors:** Oleg I. Yakhin, Aleksandr A. Lubyanov, Ildus A. Yakhin, Patrick H. Brown

**Affiliations:** ^1^Institute of Biochemistry and Genetics, Ufa Scientific Center, Russian Academy of SciencesUfa, Russia; ^2^R&D Company Eco PrirodaUlkundy, Russia; ^3^Department of Plant Sciences, University of California, DavisDavis, CA, USA

**Keywords:** biostimulants, mode of action, definition, classification, regulation, concepts, methodology, emergent properties

## Abstract

This review presents a comprehensive and systematic study of the field of plant biostimulants and considers the fundamental and innovative principles underlying this technology. The elucidation of the biological basis of biostimulant function is a prerequisite for the development of science-based biostimulant industry and sound regulations governing these compounds. The task of defining the biological basis of biostimulants as a class of compounds, however, is made more complex by the diverse sources of biostimulants present in the market, which include bacteria, fungi, seaweeds, higher plants, animals and humate-containing raw materials, and the wide diversity of industrial processes utilized in their preparation. To distinguish biostimulants from the existing legislative product categories we propose the following definition of a biostimulant as “a formulated product of biological origin that improves plant productivity as a consequence of the novel or emergent properties of the complex of constituents, and not as a sole consequence of the presence of known essential plant nutrients, plant growth regulators, or plant protective compounds.” The definition provided here is important as it emphasizes the principle that biological function can be positively modulated through application of molecules, or mixtures of molecules, for which an explicit mode of action has not been defined. Given the difficulty in determining a “mode of action” for a biostimulant, and recognizing the need for the market in biostimulants to attain legitimacy, we suggest that the focus of biostimulant research and validation should be upon proof of efficacy and safety and the determination of a broad mechanism of action, without a requirement for the determination of a specific mode of action. While there is a clear commercial imperative to rationalize biostimulants as a discrete class of products, there is also a compelling biological case for the science-based development of, and experimentation with biostimulants in the expectation that this may lead to the identification of novel biological molecules and phenomenon, pathways and processes, that would not have been discovered if the category of biostimulants did not exist, or was not considered legitimate.

## Introduction

The regulation of plant growth and the development and alleviation of the negative effects of environmental stresses during ontogenesis, are important factors determining the productivity of cultivated plants. While it is well recognized that biotic and abiotic stress prevents essentially all crop systems from achieving their yield potential, current understanding of the mechanisms involved, and the strategies to mitigate these effects are limited. Abiotic stresses may be prevented by optimizing plant growth conditions and through provision of water and nutrients and plant growth regulators (PGRs—auxins, cytokinins, gibberellins, strigolactones, brassinosteroids). In addition to these traditional approaches, biostimulants are increasingly being integrated into production systems with the goal of modifying physiological processes in plants to optimize productivity. Plant biostimulants based on natural materials have received considerable attention by both the scientific community and commercial enterprises especially in the last two and a half decades (Crouch and van Staden, 1993a; Herve, 1994; Zhang and Schmidt, 1999; Maini, 2006; Khan et al., 2009; Apone et al., 2010; Craigie, 2011; Sharma et al., 2014; Brown and Saa, 2015; Du Jardin, 2015; Yakhin et al., 2016a). Biostimulants offer a potentially novel approach for the regulation/modification of physiological processes in plants to stimulate growth, to mitigate stress-induced limitations, and to increase yield. In the following review, we do not attempt to discern if the effects of biostimulants on plant productivity is a direct response of plants or soils to the biostimulant application or an indirect response of the biostimulant on the soil and plant microbiome with subsequent effects on plant productivity. Ultimately discerning if biostimulant effects are direct or microbially mediated will be critical to the development of this technology. The general goals of the current review are to provide a comprehensive analysis of the current situation in the field of biostimulants and to develop a science-based theoretical foundation for the conceptualization, classification, and practical application of these materials. A focus of this review is to understand and define the appropriate place of biostimulants among other agricultural products such as plant protection compounds and fertilizers, and to consider the unique attributes of complex, multi-component biostimulants. The structure of the review is based on the consideration of biostimulants in terms of their action on different regulatory and functional systems of plants (signaling, metabolism, uptake, and transport mechanisms, etc.) using both conceptual and methodological approaches. The overarching objective of the work is to highlight innovative concepts and to establish a scientific framework for future development of biostimulant science.

## General concepts and methodology

To understand the development of biostimulant science, several seminal publications warrant discussion. To our knowledge, the first discussion of “biogenic stimulant” theory can be attributed to Prof. V.P. Filatov and was started in 1933 in the USSR (Filatov, 1944, 1951a,b; Gordon, 1947; Sukhoverkhov, 1967). Filatov proposed that biological materials derived from various organisms, including plants, that have been exposed to stressors could affect metabolic and energetic processes in humans, animals, and plants (Table 1). Blagoveshchensky (1945, 1955, 1956) further developed these ideas with specific reference to their application for plants, considering biogenic stimulants as “organic acids with stimulating effects due to their dibasic properties which can enhance the enzymatic activity in plants.” Filatov's concept (1951b), was, however, not limited to these compounds alone (Filatov, 1951b). Herve's (1994) pioneering review provides the first real conceptual approach to biostimulants. Herve suggests the development of novel “bio-rational products” should proceed on the basis of a systemic approach founded in chemical synthesis, biochemistry, and biotechnology as applied to real plant physiological, agricultural, and ecological constraints. He suggests these products should function at low doses, be ecologically benign and have reproducible benefits in agricultural plant cultivation. Zhang and Schmidt (1999) emphasized the need for comprehensive and empirical analysis of these products with particular emphasis on hormonal and antioxidant systems as the basis for many important benefits of biostimulants. They discuss the concept of biostimulants as “pre-stress conditioners,” their effects being manifested in improved photosynthetic efficiency, reduction of spread and intensity of some diseases and in better yields. Basak (2008) initiated the systematic discussion on biostimulants and created the conceptual preconditions for the formation of present biostimulant science while Du Jardin (2012, 2015) provided the first in-depth analysis of plant biostimulant science with an emphasis on biostimulant systematization and categorization on the basis of biochemical and physiological function and mode of action and origin. Du Jardin's (2015) analysis and categorization was influential in informing the development of subsequent legislation and regulation in the European Union.

**Table 1:** Terminology in the biostimulant field: Evolution and diversity of concepts^*^.

**Terms, variants, and synonyms of the term of “biostimulant”**	**Original definitions and hypotheses Translation from Russian publications into English is verbatim (word for word, literatim)**	**References**
Biogenic stimulators	“Every living tissue (human, animal and plants), when exposed to unfavorable, but non-lethal conditions, undergoes biochemical restructuring with the formation in it of special substances which are biogenic stimulators of non-specific nature, stimulating the life reactions of the organism, in which they introduced in, one way or another.”	Filatov, 1951a
Biogenic stimulants	“1. Organisms, either animal or plant, when exposed to such environmental factors that complicate their lives, are subjected to biochemical restructuring. Consequently, there are formed substances that stimulate biochemical processes in these tissues. These substances which help the tissues to preserve life under adverse conditions, are named stimulants of biological origin (biogenic stimulators).” “2. Biogenic stimulators, injected one way or another in any organism activates vital processes in it. By strengthening metabolism, thus they increase physiological functions of the organism. In this manner biogenic stimulants increase the organism's resistance to pathogenic factors and enhance its regenerative and absorbable properties, which facilitates recovery.” “3. Biogenic stimulators emerge as a result of biochemical restructuring, and in whole living organisms subjected to non-lethal but unfavorable external or internal environmental conditions.” “4. Environmental factors that cause the emerging of biogenic stimulants in the organism or in tissues separated from it, can be diverse.” “5. The emergence of biogenic stimulants under the influence of unfavourable factors of the environment is a common law for all wildlife. Biogenic stimulators are formed wherever there is a adaptation to new conditions of existence and the struggle for life.” “6. Biogenic stimulators accumulate in tissues and organisms when exposed to such external and internal factors that lead to the disruption of their normal metabolism, and are chemically products of disturbed metabolism.” “7. Biogenic stimulants act on the whole organism. This explains the breadth of the range of their action on the organism.” “8. The action of biogenic stimulators is expressed in changing of metabolic and energetic processes of organism.”	Filatov, 1951b
Biogenic stimulants	“Substances which are produced in living tissues when using the method of Filatov following a series of disturbances of normal metabolism for the organism (according to Filatov - resistance factors), that have a stimulating effect on various processes in the organism.” [sic] “Biogenic stimulators can not substitute for fertilizer.”	Blagoveshchensky, 1956
Organic Biostimulant	“These compounds increase plant growth and vigor through increased efficiency of nutrient and water uptake. Definitions for biostimulants vary greatly and there are still some arguments surrounding these compounds. However, they are defined as non-fertilizer products which have a beneficial effect on plant growth. Many of these biostimulant materials are natural products that contain no added chemicals or synthetic plant growth regulators.”	Russo and Berlyn, 1991
Biostimulators	“Materials of little or no fertilizer value that accelerate plant growth, usually when used at low concentrations.”	Goatley and Schmidt, 1991
Biostimulants	“Plant hormone-containing substances that can stimulate growth when exogenously applied.”	Schmidt, 1992
Allelopathic Preparation	“Multi-component balanced systems of biologically active substances of metabolic origin on the basis of plant raw materials with a broad spectrum of biological activity.”	Naumov et al., 1993
Biostimulants	A subgroup of plant growth regulators but are quite different from nutritional additives. … It is proposed to limit the use of the term biostimulant to products aimed at improving yield through various metabolistics pathways.	Herve, 1994
Biostimulants	“Products that are nonnutritive promoters of growth. Growth can be promoted by stimulating nutrient uptake, chelating nutrients, providing plant growth hormones, or enhancing plant hormonal activity. Biostimulants that contain plant growth hormones can be produced synthetically or obtained from natural plant extracts.”	Elliott and Prevatte, 1996
Biostimulant	“Materials that, in minute quantities, promote plant growth.”	Zhang and Schmidt, 1999
Biostimulant	“An ambiguous term used to encompass non-nutritional growth-promoting substances such as microbes, plant growth hormones, soil conditioners and microbe energy sources.”	McCarty, 2001
Plant Strengtheners	“Products intended to protect plants against harmful organisms by stimulating defence mechanisms in the plant or by competing with harmful organisms for space and nutrients in the phyllosphere or rhizosphere.”	(Anonymous 2001) quoted by Sharma K. et al., 2012
Biostimulant (Positive Plant Growth Regulator), (Metabolic Enhancer)	An organic material that, when applied in small quantities, enhances plant growth and development such that the response cannot be attributed to application of traditional plant nutrients. … If applied before stress occurs, biostimulants can help plants tolerate stress.	James Beard from Schmidt et al., 2003
Biostimulants	“Natural or synthetic products of either mineral or organic composition that by their mode of action positively contribute to crop nutrition and the development of healthy plants.”	(S.D. Hankins, personal communication) Dixon and Walsh, 2004
Metabolic Enhancer	“Non-mineral substances that, when exogenously applied in very small quantities, stimulate the metabolic activities in plants.”	Doak et al., 2005
Biostimolanti - Biostimulants	“Products that brings to other fertilizer and/or to the soil and/or to the plant, substances that favour or regulate the absorption of the nutrients or correct some physiological anomalies.” “It must be remind that the biostimulant activity does not have to derive from the addition of phytohormones (Attached 6, codicil 4.1). In other words, a fertilizer with “biostimulant activity” must contribute positively to the improvement of the nutrition and the development of the plants, independently from the presence of the nutrients, with the exclusion of the phytohormones, whose presence is prohibited, and of the products with declared and specific phytosanitary function.”	Ciavatta and Cavani, 2006
Biostimulants	“This term commonly identifies formulations supporting the plant in the improvement of its performances without using synthesis hormones.”	Tagliavini and Kubiskin, 2006
Organic Biostimulant	“Other than the direct influence on the nitrogen balance in plants, Siapton acts also indirectly improving the activity of some enzyme systems and regulating some plant growth regulators (PGR) functions and biochemical processes. Moreover, Siapton makes easier the absorption and the transport of the nutritional macro- and micro-elements. These statements allow to define the product as a real “organic biostimulant” and natural nutritional equilibrator for plants, more than a simple foliar organic fertilizer.”	Maini, 2006
Biostimulants	“Product generally of organic nature which increase the soil microbial activity and/or plant growth.”	Nardi et al., 2006
Biostimulants	“Single compounds or mixtures of compounds which mitigate the effects caused by abiotic stress agents, through the induction of innate plant defense responses.”	Apone et al., 2006
Biostimulant Plant Growth Promoters Growth-Promoting PGRs	“Organic or hormone-containing compounds derived from natural products which can stimulate plant growth and development without causing known harm to the environment.”	Huang, 2007
Biostimulators	“Compounds of biological origin and should act by increasing natural capabilities of plants to cope with stresses. Biostimulators do not act neither as nutrients nor affect directly the stress factors making them less harmful for plants.”	Gawrońska, 2008
Biostimulators	“Agents which at very low concentrations improve the basic biochemical processes in plants and soil, and thereby improve the growth and development of plants, and increase their resistance to stress. Biostimulators are not a substitute for fertilizers, manure or other sources of mineral nutrients.”	Anonymous, cited by Basak, 2008
Biostimulators	“Innovative natural stimulators of plant growth and development, obtained from biological raw materials, and having a hormone- or fertilizer-like mode of action.”	Anonymous, cited by Basak, 2008
Biofertiliser/Biostimulant	“Are organic products composed of peptides, amino acids, polysaccharides, peptides, humic acids, and/or phytohormones, etc. for immediate uptake and availability within the plant. Their absorption does not depend on the photosynthetic activity as they are directly absorbed by the plant, resulting in lower energy consumption. The aim of these products is not to supply nutrition, but rather to favour and stimulate the metabolism of the plant, decrease plant stress, etc.”	Parrado et al., 2008
Organic Biostimulants	“Non-nutrient containining products which have beneficial effect on plant growth. Such products consist of humic acids, marine algae (sea weed) extracts, non-hormonal plant metabolites and vitamins.”	Kumar and Shivay, 2008
Phytostimulator	“Microorganism with the ability to produce or change the concentration of growth regulators such as indole acetic acid, gibberellic acid, cytokinins and ethylene.”	Martínez-Viveros et al., 2010
Agricultural Biostimulants	“Include diverse formulations of compounds, substances and other products that are applied to plants or soils to regulate and enhance the crop's physiological processes, thus making them more efficient. Biostimulants act on plant physiology through different pathways than nutrients to improve crop vigour, yields, quality and post-harvest shelf life/conservation.”	European Biostimulants Industry Council EBIC, 2011b
Biostimulators	“They mean inorganic and organic substances or its mixtures positively affecting plant development or other physiological processes in plants. One of the requirements for biostimulators is that they pose no risk for human, animal, or natural environment due to its application.”	Smoleń S, 2012
Biostimulants	“Materials that are neither a fertilizer nor a pesticide, but when applied to a plant will enhance their health, growth, and protection.”	Banks and Percival, 2012
Plant Biostimulants	“Substances and materials, with the exception of nutrients and pesticides, which, when applied to plants, seeds or growing substrates in specific formulations, have the capacity to modify physiological processes of plants in a way that provides potential benefits to growth, development and/or stress response.”	Du Jardin, 2012
Antitranspirant	“Indicates the overall effect on the plant, the chemical compounds and underlying mechanisms are very diverse. Some of the compounds have physical effects at the surface and/or within the plant organs, others are regulators of the leaves openings diffusing water vapor, called stomata.”	Du Jardin, 2012
Metabolic Antitranspirants	“Stomatal regulators, acting on the complex hormonal control of the highly specialized cells bordering the stomatal pore (guard cells).”	Du Jardin, 2012
Plant Biostimulants	“Contain substance(s) and/or micro-organisms whose function when applied to plants or the rhizosphere is to stimulate natural processes to enhance/benefit nutrient uptake, nutrient efficiency, tolerance to abiotic stress, and crop quality. Biostimulants have no direct action against pests, and therefore do not fall within the regulatory framework of pesticides.”	European Biostimulants Industry Council EBIC, 2012
Biostimulant	“Material that, when applied to a plant, seed, soil or growing media - in conjunction with established fertilization plans, enhances the plant's nutrient use efficiency, or provides other direct or indirect benefits to plant development or stress response.”	Beaudreau, 2013, Biostimulant Coalition
Biostimulants	“Compounds that produce non-nutritional plant growth responses and reduce stress by enhancing stress tolerance. This is in contrast to fertilizers, which produce a nutritional response. Many important benefits of biostimulants are based on their ability to influence hormonal activity.” “Compounds in biostimulants can alter the hormonal status of a plant and exert large influences over its growth and health.”	Daniels, 2013
Plant Strengthener (Biostimulant)	A class of “borderline” products used in agriculture in some member states … which act only on plant strength without direct effects against pests and have no main nutritional activity, … enhance the resistance of plants to harmful organisms and protect plants against non-parasitic impairments.”	Torre et al., 2013
Biostimulant	“Products which, alone or mixed with other fertilizers, contribute to improve plant growth by exploiting different physiological processes.”	Migliore et al., 2013
Bio-Stimulatory Bio-stimulatory Agent	“The term “bio-stimulatory” means according to the invention, if not otherwise specified, an activity or efficacy which stimulates, increases or improves many different processes in the plant or plant parts, such as improved generation of growth promoting substances like sugars and amino acids, improved adequate supply of cells with available nutrients and growth regulators, enhanced cell metabolism, improved cell decontamination, enhanced immune defense, promotion of growth and yield, induction of systemic acquired resistance (SAR), inhibition of growth and yield of competing plants (allelopathy). The bio-stimulatory activity can be caused by agents, plant extracts and compositions including metabolic compounds synthesized by the plant to be protected after induction of their synthesis by said bio-stimulatory agent. A “bio-stimulatory agent” according to the invention is a biological plant protecting agent as specified above, which shows the above-specified bio-stimulatory properties in a plant treated with this agent *in vitro* and/or *in vivo*.”	Pretorius, 2013
Biostimulant	“Is an organic material that, when applied in small quantities, enhances plant growth and development such that the response cannot be attributed to the application of traditional plant nutrients.”	Sharma et al., 2014
Biostimulant Microorganisms	“Both biocontrol microorganisms (BCMs) and plant growth-promoting microorganisms (PGPMs) can be defined as “biostimulant microorganisms,” able to foster plant growth and defence against pathogens throughout the crop life cycle, from seed germination to plant maturity.”	Sofo et al., 2014
Phytostimulators Biostimulators	“This category includes microorganisms that promote plant growth usually by hormonal action.”	Aguado-Santacruz et al., 2014
Plant Biostimulant	“Any substance or microorganism, in the form in which it is supplied to the user, applied to plants, seeds or the root environment with the intention to stimulate natural processes of plants benefiting nutrient use efficiency and/or tolerance to abiotic stress, regardless of its nutrients content, or any combination of such substances and/or microorganisms intended for this use.”	Traon et al., 2014
Biostimulants	“Are extracts obtained from organic raw materials containing bioactive compounds.”	Bulgari et al., 2015
Biostimulants	“Are materials, other than fertilizers, that promote plant growth when applied in small quantities. These environmental friendly and natural substances promote vegetative growth, mineral nutrient uptake and tolerance of plants to abiotic stresses.”	Chojnacka et al., 2015
Biostimulant	“Any substance or microorganism applied to plants with the aim to enhance nutrition efficiency, abiotic stress tolerance and/or crop quality traits, regardless of its nutrients content. By extension, plant biostimulants also designate commercial products containing mixtures of such substances and/or microorganisms.”	Du Jardin, 2015
Biostimulants	“Are substances or materials, with the exception of nutrients and pesticides, which, when applied to plants, seeds, or growing substrates in specific formulations, have the capacity to modify physiological processes in plants in a way that provides potential benefits to growth, development, or stress response.”	Halpern et al., 2015
Biostimulant	“Refers to a compound or composition that is neither a fertilizer nor pesticide, but which when applied to a plant will enhance the health and growth of a plant.”	Lovatt, 2015
Biostimulants	“Products mostly based on natural raw materials, used in the ultra-small and small doses for modification of physiological and biochemical plant processes with the aim of more complete realization of genetic potential of their productivity due to changes in hormonal status, activation of metabolic processes, increase of efficiency of nutrition, stimulation of growth, development and strengthening the ability to withstand to the negative effects of various stress factors.”	Yakhin et al., 2016a

* *The definitions are provided as exact quotes from the primary sources without correction of spelling or grammar*.

The study and development of biostimulants has been approached utilizing a wide range of methodological approaches including chemical and non-chemical characterization of composition (Crouch and van Staden, 1993b; Yakhin et al., 2005; Parrado et al., 2008; Sharma et al., 2012a,b; Ertani et al., 2013a,b; Aremu et al., 2015a,b), plant growth and yield studies (Khan et al., 2009; Kunicki et al., 2010; Parađiković et al., 2011; Zodape et al., 2011; Yakhin et al., 2012, 2016b; Chbani et al., 2013; Kurepin et al., 2014; Colla et al., 2015; Saa et al., 2015; Tandon and Dubey, 2015; Tian et al., 2015), application of the so-called *-omics* strategies with variations, including microarray and physiological analysis (Jannin et al., 2012, 2013), transcriptome (Wilson et al., 2015; Goñi et al., 2016), genomic (Santaniello et al., 2013), phenomic and molecular (Petrozza et al., 2014), proteomic (Martínez-Esteso et al., 2016), chemical and metabolomic (Ertani et al., 2014). Ultimately, the integrative synthesis of results from multiple methodologies, particularly when integrated with the most relevant—*omic* technology, “agronomics,” will be required if the science and legitimacy of plant biostimulants is to advance.

Several significant scientific meetings in the field of biostimulants have been held over the past ten years and have contributed greatly to our understanding of conceptual and methodological development of the biostimulant theory: “Biostimolanti in agrocoltura” (Italy, 2006), “Biostimulators in Modern Agriculture” (Poland, 2008), “Biostimulants and Plant Growth” (Belgium, 2014), among others. Of particular significance were the first (France, 2012) and the second (Italy, 2015) World Congresses on the “Use of Biostimulants in Agriculture” which were valuable in highlighting the development of novel concepts and methodology as applied to biostimulants. While many of the following papers are not published in a peer-reviewed format, they do represent important advances in this field. Dumas et al. (2012), for example, proposed a multi-part approach to study biostimulants based on large-scale genomic approaches and high-throughput screening tests with genetically-modified reporter plants. Others suggested that biostimulant mode of action can be best determined using molecular microarray analysis to identify gene changes in transcript levels (Gates et al., 2012). This approach has the potential to reveal biostimulant activated signaling pathways involved in the stimulation of plant response. Microarray analysis is not, however, adequate and must be supplemented with carefully conducted field testing or high throughput plant phenotyping (Summerer et al., 2013). The complexity of known biostimulant response, the dependency of crop environment and the diversity of biostimulant products demands the application of novel statistical approaches not commonly used in agronomic research (Sleighter et al., 2015). The principle espoused by Sleighter et al. (2015) is based on the identification of a subset of molecular markers that represent the active ingredients in complex biostimulants and then to correlate these markers with observations of plant response. Chemical genomics that utilizes small molecules to perturb target protein function is a useful strategy for biostimulant discovery as it overcomes constraints imposed by traditional molecular approaches that often fail due to gene redundancy and loss-of-function lethality. Botta et al. (2015) proposed probing the function of biostimulants using an enantiomeric analysis of active compounds in the biostimulant coupled with a proteomic profiling approach. In contrast, Conan et al. (2015) proposed identification of the bioactive compounds responsible for the plant growth response by means of a metabolomic profiling of biostimulant products and analysis of their physiological effects through transcriptomic and metabolomic strategies. Such methodology allows the determination of metabolite pathways affected by biostimulants as well as providing insight into gene regulation. To integrate the diversity of methodologies available Santaniello et al. (2015) emphasizes the need to use bioinformatics strategies to analyse similarities and differences in procedures of ingredient extraction and biostimulant formulation in terms of molecular plant responses. This integrative concept can be used to derive new technologies and novel biostimulant products through the identification of new target genes, enzymes and metabolites.

While the development of robust, multi-faceted approach to the analysis of biostimulant composition and function will greatly aid in the development of this field, all advances must ultimately be interpreted in the context of plant response. The complexity of plant response to the environment is daunting and was elegantly highlighted by Krouk (2015) who demonstrated that root response to nitrogen in the environment is mediated by combinations of signaling molecules and nitrogen sources in a manner that cannot be predicted by exposure to single compounds provided individually (Krouk, 2016; Krouk et al., 2009, 2010, 2011). Inevitably, as our understanding of the molecular networks that control plant growth improves our ability to predict plant response to biostimulants under specific environmental conditions, will improve. Only through a combination of methodologies will progress in biostimulant research be possible.

## Terminology and definitions

The development of plant biostimulant science, as well as the principles governing its legislation in the context of the existing legal frameworks of plant protection products and fertilizers, requires the development of a clear definition of term “biostimulant.” Currently, the term “biostimulant” is poorly defined and includes many products that have variously been described as biogenic stimulants, metabolic enhancers, plant strengtheners, positive plant growth regulators, elicitors, allelopathic preparation, plant conditioners, phytostimulators, biofertilisers, or biofertiliser/biostimulant (Table 1). One area of significant challenge is evoked in the question “are biostimulants PGRs?” Historically, biostimulants have been considered as a subgroup of growth regulators (Herve, 1994), as plant growth regulators (Huang, 2007), and as subgroup of bioregulators (Basak, 2008). “From a legal point of view, biostimulants can contain traces of natural plant hormones, but their biological action should not be ascribed to them, otherwise they should be registered as plant growth regulators” (Bulgari et al., 2015). Likewise, biostimulants cannot by definition be pesticides or fertilizers (Russo and Berlyn, 1991; Karnok, 2000; Hamza and Suggars, 2001; Banks and Percival, 2012; Du Jardin, 2012; Torre et al., 2013, 2016).

A concise and biologically meaningful definition of biostimulants has eluded researchers and regulators for many years. Table 1 presents a chronological evolution of concept of the term biostimulant. While several of biostimulant definitions presented are useful in their breadth, many of them have significant limitations and are overly generic, while several do not exclude possible effects of nutrients contained within any putative biostimulant product. In practice, biostimulants may deliberately include nutrients for regulatory approval as fertilizers and on occasions the included nutrients or hormones may be responsible for the perceived agronomic benefit. Given the state of public mistrust of many “biostimulant” products, it is necessary to provide a definition of biostimulants that explicitly denies the use of this term for products that do not have biological efficacy or have efficacy only by virtue of the inclusion of known plant hormones or nutrients.

While the adoption of a definition of biostimulants for regulatory purposes is important, any definition of biostimulant should also be based on scientific principles. Several concepts have been proposed to define plant biostimulants. Basak (2008), proposed that biostimulants could be classified depending on the mode of action and the origin of the active ingredient while Bulgari et al. (2015), proposed that “biostimulants should be classified on the basis of their action in the plants or, on the physiological plant responses rather than on their composition.” Du Jardin (2015), however, has emphasized the importance of the final impact on plant productivity when he suggests that “any definition of biostimulants should focus on the agricultural functions of biostimulants, not on the nature of their constituents nor on their modes of actions.” The term “plant productivity” is used here to describe any improvement in plant yield or quality or increased efficiency of production. These concepts reflect important differences in approaches to providing a definition of biostimulants as a discrete category of agricultural products. Thus, biostimulants could be defined by their demonstrated mode of action and origin, or solely by their demonstrated beneficial impact on plant productivity. The challenges in developing a definition are also complicated by the multi-component and largely undefined composition of many biostimulant products and the possibility that the activity of a biostimulant may not be explained by the presence of any individual constituent, but is a result of the interaction of many constituents in the product.

On this basis two approaches to the definition of complex biostimulants emerge. The first is based on the possibility that the biostimulant contains within it, previously unrecognized molecules that are the sole and discrete cause of the observed improvement in plant productivity. This concept emphasizes both the need for clear demonstration of plant productivity benefits and the unknown nature of the mode of action. Thus, a biostimulant could be defined as “a formulated product that improves plant productivity by a mechanism of action that is not the sole consequence of the presence of known essential plant nutrients, plant hormones, plant growth regulators or plant protective compounds.” By this definition, once the primary biological mechanism of biostimulant function has been identified it should henceforth, be subject to classification on the basis of that functional component.

The majority of biostimulants in use today are complex mixtures of chemicals derived from a biological process or extraction of biological materials. The complexity of these mixtures is often considered to be essential to the performance of the biostimulant, and biostimulants may have properties of the whole, that cannot be fully elucidated by knowing the characteristics of the separate components or their combinations. This theory of complexity or “emergence” was described by Mayr (1982), who argued that in many biological systems “the properties of the whole cannot be fully elucidated by knowing the characteristics of the separate components or their combinations.” “The term emergence describes the onset of novel properties that arise when a certain level of structural complexity is formed from components of lower complexity. In the last few decades, emergence has been discussed in a number of different research fields, such as cybernetics, theory of complexity, artificial intelligence, non-linear dynamics, information theory, and social systems organization” (Luisi, 2002). “Emergence” and “emergent properties” are thus closely related with the notion of the “systems biology” (Luisi, 2002; Johnson, 2006; Korosov, 2012; Lüttge, 2012; Bertolli et al., 2014). Emergence was described by Johnson (2006) as “unexpected behaviors that stem from interaction between the components of an application and their environment,” “there is, however, considerable disagreement about the nature of ‘emergent properties.’ Some include almost any unexpected properties exhibited by a complex system. Others refer to emergent properties when an application exhibits behaviors that cannot be identified through functional decomposition. In other words, the system is more than the sum of its component parts” (Johnson, 2006).

Thus, a biostimulant could also be defined as “a formulated product of biological origin that improves plant productivity as a consequence of the emergent properties of its constituents.”

To our knowledge, however, there have been no clear demonstrations that any biostimulant exhibits truly emergent properties. This is not however a unique challenge and all “biological systems are extremely complex and have emergent properties that cannot explained, or even predicted, by studying their individual parts” (Van Regenmortel, 2004). Emergent properties have been demonstrated in the networks of biological signaling pathways (Bhalla and Iyengar, 1999); in system-level study of traditional Chinese medicine (Chen et al., 2014), and in microbial communities (Wintermute and Silver, 2010; Chiu et al., 2014). To adequately explain the biological complexity present in plants and their interactions with the environment, Lüttge (2012) and Bertolli et al. (2014) emphasize that classic reductionist biology/chemistry is indeed insufficient.

While the two theoretical definitions provided in this section share a requirement that the mode of action is unknown, they differ in the core assumption that biostimulant function is a consequence of the discrete components in the biostimulant or as a consequence of the “emergent” properties of the biostimulant as a whole. Each of these definitions is also incomplete in that it is certainly possible that a biostimulant may contain several molecules that act synergistically while not being truly “emergent,” and it is indeed possible and indeed likely, that even if a biostimulant is demonstrated to have emergent properties, that not all components of that biostimulant are required for that property to be expressed.

We propose, therefore, a definition of a biostimulant that integrates these two concepts. Thus, a biostimulant is defined here as:

“a formulated product of biological origin that improves plant productivity as a consequence of the novel, or emergent properties of the complex of constituents, and not as a sole consequence of the presence of known essential plant nutrients, plant growth regulators, or plant protective compounds.”

Consistent with this definition, the ultimate identification of a novel molecule within a biostimulant that is found to be wholly responsible for the biological function of that biostimulant, would necessitate the classification of the biostimulant according to the discovered function.

## Classification

A review of the history of biostimulants and related products provides insight into the diversity of these products and the development of this field of study. The evolution of biostimulant classifications as described by various authors is presented in the Table 2. To the best of our knowledge, one of the first attempts to categorize biostimulants was provided by Filatov (1951b) when 4 groupings of biogenic stimulants were suggested. Karnok (2000) compiled a list of 59 materials presenting in 15 biostimulants; Ikrina and Kolbin (2004) systematized patent literature and specified 9 categories of natural raw materials used to derive biostimulants; Basak (2008) suggested that biostimulants could be grouped on the basis of single or multicomponent formulations and classified on the origin of the active ingredient, and the mode of action of the active ingredient. Du Jardin (2012) developed a scientific rationale of classification considering 8 categories of biostimulants and subsequently reduced this list to 7 categories (Du Jardin, 2015). Du Jardin (2012) was explicit in his exclusion of microorganisms from his categorization primarily to avoid conflict with existing categorization of microorganisms as biopesticides and sources of plant hormones. Later Bulgari et al. (2015) proposed a biostimulant classification on the basis of their mode of action rather than on their composition.

**Table 2:** **Proposed Biostimulant Categories**.

	**Filatov, 1951b**	**Ikrina and Kolbin, 2004**	**Kauffman et al., 2007**	**Du Jardin, 2012**	**Calvo et al., 2014**	**Halpern et al., 2015**	**Du Jardin, 2015**	**Torre et al., 2016**
1	Carboxylic fatty acids (oxalic acid and succinic acid)	Microorganisms (bacteria, fungi)	Humic substances	Humic substances	Microbial inoculants	Humic substances	Humic and fulvic acids	Humic substances
2	Carboxylic fatty hydroxy acids (malic and tartaric acids)	Plant materials (land, freshwater and marine)	Hormone containing products (seaweed extracts)	Complex organic materials	Humic acids	Protein hydrolysate and amino acid formulations	Protein hydrolysates and other N-containing compounds	Seaweed extracts
3	Unsaturated fatty acids, aromatic and phenolic acids (cinnamic and hydroxycinnamic acids, coumarin)	Sea shellfish, animals, bees	Amino acid containing products	Beneficial chemical elements	Fulvic acids	Seaweed extract	Seaweed extracts and botanicals	Hydrolyzed proteins and amino acids
4	Phenolic aromatic acids containing several benzene rings linked via carbon atoms (humic acids)	Humate- and humus-containing substances		Inorganic salts (such as phosphite)	Protein hydrolysates and amino acids	Plant-growth-promoting microorganisms (including mycorrhizal fungi)	Chitosan and other biopolymers	Inorganic salts
5		Vegetable oils		Seaweed extracts	Seaweed extracts		Inorganic compounds	Microorganisms
6		Natural minerals		Chitin and chitosan derivatives			Beneficial fungi	
7		Water (activated, degassed, thermal)		Antitranspirants			Beneficial bacteria	
8		Resins		Free amino acids and other N-containing substances				
9		Other raw materials (oil and petroleum fractions, shale substance)						

Many biostimulant products have been classified into completely divergent groups and categories of function, use, and type of activity (Tables 3, 4). For example, humate-based products are often described as soil health amendments while plant growth promoting rhizobacteria (PGPRs) could be categorized as biofertilizers, phytostimulators, and biopesticides (Martínez-Viveros et al., 2010; Bhattacharyya and Jha, 2012). Du Jardin (2015) has proposed that biofertilisers are a subcategory of biostimulants. Seaweed extracts have been considered as biofertilizers (Zodape, 2001) and microorganisms have also been described as biofertilizers (Vessey, 2003; Fuentes-Ramirez and Caballero-Mellado, 2006; Roy et al., 2006; Malusá et al., 2012; Bhardwaj et al., 2014; Malusá and Vassilev, 2014). Some inorganic elements or small molecules that are not known to be essential may also be classified as biostimulants if evidence of plant growth promotion is available (Michalski, 2008; Kleiber and Markiewicz, 2013; Radkowski and Radkowska, 2013). Thao and Yamakawa (2009), for example, consider phosphites to be biostimulants since plant response to phosphites frequently cannot be explained as a consequence of the known anti-fungal function of these molecules. While the categorization of biostimulants by their origin does not, *a priori*, provide information on their mode of action this categorization may still be a useful tool to aid in the process of discovery and facilitate comparison between similar products.

**Table 3:** **Examples of different terminology used for commercial biostimulants**.

**Preparations**	**Source, composition**	**Found in the literature related terms**	**References**
Actiwave®	*Ascophillum nodosum*	Metabolic enhancer	Spinelli et al., 2010
		Biostimulant	Vernieri et al., 2006; Ferrante et al., 2013
Agrispon®	Natural plant extract	Biostimulant	Rouse, 1984
		Bioregulator, Biostimulant	Dubravec et al., 1995
		Biostimulator	Michalski, 2008
Aminoplant (Siapton®)	Epithelial tissues (natural substances animal origin)	Organic biostimulant, Soil fertilizer	Maini, 2006
		Biostimulant	Betti et al., 1992; Mladenova et al., 1998; Apone et al., 2006; Cambri et al., 2008; Kunicki et al., 2010
		Fertilizer	Mladenova, 1978
Asahi SL	Sodium para-nitrophenolate, sodium ortho-nitrophenolate, sodium 5-nitroguaiacolate	Biostimulant	Basak, 2008; Przybysz et al., 2014
(Atonik)		Bioregulator	Michalski, 2008
Bio-Algen®	*Phaeophyceae*	Biostimulator, Bioregulator	Basak, 2008
Biozyme®	*Ascophyllum nodosum* (GA_3_+IAA+zeatin+ chelated micronutrients)	Biostimulant	Tandon and Dubey, 2015
		Bioregulator	Belakbir et al., 1998; Ruiz et al., 2000
ComCat®	*Lychnis viscaria*	Plant growth regulator, biostimulant,	Van der Watt and Pretorius, 2013
Ergostim®	L-cysteine and folic acid derivative	Plant growth regulator, Biostimulant	Cutler and Cutler, 2004
		Biostimulant	Gupta and MacLeod, 1982; Sanders et al., 1990; Kinnersley, 1993
		Bioregulator, Biostimulant	Dubravec et al., 1995
Fantac (Quantum)	Mixture of 5% N-Acetyl thiazolidine carboxylic acid (N-ATCA) and 0.1% folic acid	Biostimulant, growth promoter	Srivastava et al., 2008, 2010
FOLIAR (Macro-Sorb Foliar)	A complex water soluble solution derived from the enzymatic hydrolysis of animal membranes	Biofertilizer	Aylward, 2005
		Biostimulant	Kauffman et al., 2007
Goëmar BM 86®	*Ascophyllum nodosum*	Fertilizer, Biostimulator, Bioregulator	Basak, 2008
		Fertilizer	Craigie, 2011
Kelpak®	*Ecklonia maxima*	Biostimulant	Arthur et al., 2013; Stirk et al., 2014
		Biostimulator, Bioregulator	Basak, 2008
		Plant growth regulator, bioregulator	Michalski, 2008
		Fertilizer	Dhargalkar and Pereira, 2005
		Plant growth stimulant	Khan et al., 2009
Maxicrop®	*Ascophyllum nodosum*	Biostimulator, Bioregulator	Basak, 2008
		Fertilizer	Dhargalkar and Pereira, 2005
		Plant growth stimulant	Khan et al., 2009
Seasol (Agrikelp)	*Durvillea potatorum*	Plant growth stimulant	Khan et al., 2009
		Liquid organic fertiliser	Tay et al., 1987; Kurepin et al., 2014
		Biostimulant	Sharma et al., 2014
Stifun^*^	The complex of biologically active substances of natural origin	Bioregulator	Yakhin et al., 2006, 2007, 2011a
		Plant growth regulator	Yakhin et al., 2011b, 2012, 2013
		Biostimulant	Yakhin et al., 2014, 2016a,b
SM3 (Sea Magic 3)	*Laminariaceae* and *Fucaceae species*	Biostimulator, Bioregulator	Basak, 2008
Tytanit®	Titanium	Biostimulant	Basak, 2008
		Fertilizer	Kleiber and Markiewicz, 2013
Wuxal®-Ascofol	*Ascophyllum nodosum*	Biostimulator, Bioregulator	Basak, 2008
–	moringa leaf extract	Plant growth stimulant	Yasmeen et al., 2013
		Biostimulant	Abdalla, 2013; Yasmeen et al., 2014

* *By the results of state registration tests Stifun was recommended for registration but does not registered yet*.

**Table 4:** **Biostimulants: sources, production, compositions, and activities**.

**Genus, species of organism /source of raw material**	**Methods of production**	**Methods of identification/ standardization**	**Ingredients and bioactive compounds**	**Hypothesized modes/mechanism of action**	**Biological effects**
**1**	**2**	**3**	**4**	**5**	**6**
**1. BACTERIA**					
**1. Preparations of living microorganisms:** *Aeromonas rivuli, Agromyces fucosus, Bacillus licheniformis, Bacillus megaterium, Bacillus pumilus, Bacillus safensis, Microbacterium sp., Nocardia globerula, Pseudomonas fluorescens, Pseudomonas fulva, Pseudoxanthomonas dajeonensis, Rhodococcus coprophilus, Sphingopyxis macrogoltabida, Streptomyces spp*.	Cultivation	ARISA fingerprinting, ELISA, GC-MS, Immunoblot, Most Probable Number, NMR, Spectroscopy, Molecular taxonomical characterization; Plate Count methods, Thermochemolysis, TLC.	Substances with auxin (IAA)-like bioactivity, IAA, cytokinins, betaines, gibberellins, amino acids, oligopeptides, low-molecular-weight peptides, peptidoglycans; lypopolysaccharides, melatonin.	Increase availability of nutrients in soil. Stimulate nitrogen uptake. Maintain soil fertility, nitrogen fixation, solubilize insoluble minerals through the production of organic acids. Auxin-like, gibberellin-like activity. Influence on the hormonal status of the plant. Stimulate amino acid synthesis. Increase concentrations of total carbohydrates. Increase nutrients (magnesium, nitrogen and phosphorus, etc.). Increase pigments (chlorophyll, carotenoids). Increase antioxidant substances. Stress resistance: heat, drought, wear, traffic, and/or salinity. Control fungal diseases and other physiological disorders. Activation of systemic resistance (ISR and SAR).	Increase germination rate, growth characters (length, fresh, dry weight) of shoots and roots, plant quality, productivity, yield.
**2. Preparations on the basis of non-living microorganisms and their metabolites:** *Bifidobacterium bifidus, Lactobaccillus spp., Lactobacillus acidophilus, Lactobacillus buchneri, Lactobacillus delbrueckii, Lactobacillus johnsonii, Lactobacillus murinus, Lactobacillus paraplantarum, Lactobacillus pentosus, Lactobacillus plantarum, Lactococcus tactis, Leuconostoc oenos, Propionibacterium freudenreichii, Propionibacterium pelophilus, Propionibacterium shermani, Propionibacterium spp., Propionivibrio limicola, Streptococcus spp., Streptococcus thermophilus* (*also called Streptococcus salivarius*); Bacterial cell cream from an industrial fermentation process.	Acid hydrolysis, alkali hydrolysis, cultivation, enzymatic hydrolysis, fermentation.				
**References:** Bashan, 1998; Linser et al., 2006; Borriss, 2011; Tachibana et al., 2012; Abbas, 2013; de Fretes et al., 2013; Giannattasio et al., 2013; Janas and Posmyk, 2013; Jenkins, 2014; Sofo et al., 2014; Colla et al., 2015; Spaepen, 2015.					
**2. FUNGI**					
**1. Preparations of living microorganisms:** *Glomus intraradices, Trichoderma atroviride*. **2. Preparations derived from non-living microorganisms and their metabolites:** *Candida spp., Hanseniaspora spp., Issatchenkia spp., Kloeckera spp., Kluyveromyces spp., Metschnikowia spp., Pichia spp., Saccharomyces bayanus, Saccharomyces boulardii, Saccharomyces cerevisiae, Saccharomyces exiguous, Saccharomyces pastorianus, Saccharomyces pombe, Syncephalastrum racemosum*.	Cultivation, fermentation, lyophilization.	ARISA fingerprinting, sonication and gradient flotation. NMR, bioassays, HPLC, FTIR.	Amino acids, auxin-like compounds, betaines, carbohydrates, chitosan, cytokinins, exopolysaccharides, gibberellins, IAA, melatonin, minerals, nucleic acids, oligopeptides, oligoproteins, polyglucuronic acid, proteins, siderophores, vitamins.	Increase nutrient uptake. Stimulate of nitrogen uptake. Increase enzyme activity. Influence on soil and plant metabolism. Change hormonal status of the plant. Stimulate amino acid synthesis. Increase total carbohydrates and total protein. Increase in total soluble sugars, total free amino acids, and total phenols. Increase pigments (chlorophyll, carotenoids). Increase nutrients concentrations. To induce plant defense reactions. Enhance environmental stress tolerance: drought, salinity, soil disturbance, toxic pollutants. Limit spread of disease by microbial competition. Prevent pathogen infection by eliciting resistance mechanisms such as systemic induced resistance. Reduce pathogen inoculum in the rhizosphere, thereby reduce the incidence of infection.	Increase germination rate, growth characters (length, fresh, dry weight) of shoots and roots; vegetative growth; the size of plants; the number of flowers; the number of fruits; plant quality; productivity; yield and yield components.
**References:** Xavier and Boyetchko, 2002; Adholeya et al., 2005; IJdo et al., 2010; Gandarillas Infante, 2012; Abbas, 2013; Giannattasio et al., 2013; Janas and Posmyk, 2013; Hammad and Ali, 2014; Jenkins, 2014; Sofo et al., 2014; Valepyn et al., 2014; Colla et al., 2015.					
**3. ALGAE**					
*Ascophyllum nodosum, Caulerpa scalpelliformis, Chlorella ellipsoida, Durvillea antarctica, Durvillea potatorum, Ecklonia maxima, Enteromorpha flexuosa, Fucus serratus, Fucus vesiculosus, Gelidiella acerosa, Gracilaria corticata, Gracilaria salicornia, Himanthalia elongate, Hypnea musciformis, Kappaphycus alvarezii, Laminaria digitata, Laminaria hyperborean, Macrocystis integrifolia, Macrocystis pyrifera, Padina boergesenii, Padina gymnospora, Padina pavonica, Sargassum muticum, Sargassum tenerimum, Sargassum wightii, Spirulina maxima, Ulva lactuca*.	Acid processing; acidic extraction; alkaline extraction; alkaline hydrolysis; alkaline processing; aqueous extraction; cell burst; cell rupture with high pressure treatment; cold or frozen, alkaline and water extractions; cryoprocessing; enzyme-assisted extraction (EAE); fermentation; heated alkaline hydrolysis; microwave-assisted extraction (MAE); neutral extraction; pressurized liquid extraction (PLE; also known as pressurized fluid extraction, enhanced solvent extraction, high-pressure solvent extraction, or accelerated solvent extraction techniques); supercritical fluid extraction (SFE); ultrasound-assisted extraction (UAE).	^13^CNMR, ^1^H NMR (qNMR), Bioassay, DEPT together with 2D experiments (GCOSY, GHSQC and GHMBC), ELISA, energy dispersive X-ray microanalysis (EDX), Fourier-transform infrared spectroscopy (FTIR), GC/MS, GLC, HPLC, HPLC/MS/MS, HPLC/MS, inductively coupled plasma-optical emission spectroscopy (ICP-OES), IR, LC-MS, LC-MS-MS, mass spectrometry (ESI-TOF–MS), NMR, pyrolysis gas chromatography/mass spectrometry (Py-GC/MS), scanning electron microscopy, thermogravimetry (TGA), TLC, Ultra high performance liquid chromatography–tandem mass spectrometry (UHPLC–MS/MS) analysis, X-ray microanalysis.	1-Aminocyclopropane-l-carboxylic Acid (ACC); abscisic acid (ABA); alginic acid; Auxins (IAA, IAAsp, IAAla, IAGly, IALeu, ICA, ILA, IPA, IPia, ICA, N,N-dimethyltryptamine, IAId, iso-indole, 1, 3-dione (N-hydroxy ethylphthalimide), auxin-like substances, phenyl-3-acetic acid (PAA) and hydroxyphenyl acetic acid (OH-PAA); Betaines (Glycinebetaine, γ-aminobutyric acid betaine, δ-aminovaleric acid betaine, glycinebetaine, laminine, lysinebetaine, ascophylline); Carbohydrates: 1-(2-furanyl) ethanone (mannitol), 5-methyl-2-furcarboxaldehyde (fucoidan), 2-hydroxy-3-methyl-2-cyclopenten-1-one (laminarin), diannhydromannitol (mannitol), 1,6-anhydromannopyranose and 1,6-anhydromannofuranose (mannitol); Cytokinins: zeatin (Z), dihydrozeatin (DHZ), trans-zeatin (tZR), cis-zeatin (cZR), dihydrozeatin riboside (DHZR), isopentenyladenine (iP), isopentenyladenosine (iPR), benzyladenine riboside (BAR), meta-topolin (mT), meta-topolin riboside (mTR), ortho topolin (oT), and ortho-topolin riboside (oTR), cytokinin glucosides, etc. Gibberellic acid (GA_3_); carrageenans; lipids; melatonin; minerals (Na, Ca, Cu, Fe, I, K, Mg, Na, P, S, B, Mn, Zn, Co, potassium oxide, phosphorus oxide, N, S, Cl, ${\text{HCO}}_{3}^{-}$, etc.); oligosaccharides; pepsin; phenolic compounds: eckol, phloroglucinol, etc. polysaccharides, protein, sterols: 22-Dehydrocholesterol; 24-Methylenecholesterol; 24-Methylenecycloartanol; 24-Methylenophenol; 28-Isofucosterol; 5-Dihydroergosterol; Brassicasterol; Campesterol; Cholesterol; Chondrillasterol; Clerosterol; Clionasterol; Codisterol; Cycloartenol; Decortinol; Decortinone; Desmosterol; Ergosterol; Fucosterol; Isodecortinol; Obusifoliol; Ostreasterol; Poriferastenol; Sitosterol; ß-Stitosterol; Stigmasterol; Zymosterol; Δ^4, 5^—Ketosteroids; Δ^5^—Ergostenol; Δ^7^—Ergostenol, etc.	Increase nutrient absorption and fertilizer efficiency; nutrient uptake; uptake of Cu, Ca, K and Mg; macro- and microelements content; assimilation of N, C, and S; could reduce the fertilizers. Efficient water uptake. Auxin-, Cytokinin-, Gibberellin-like activity. Modulation of phytohormones. Regulation of gene expression. Increase photosynthetic efficiency; photosynthetic pigments (chlorophyll, carotenoids); total protein concentrations; amino acid, betaines, carbohydrate content; ascorbic acid; nutrient concentrations. Increase metabolites including phenolic compounds. Up-regulation of bio-synthetic enzymes; enhance antioxidant activity. Enhance biosynthesis of non-enzymatic compounds. Delay senescence. Reduce transpiration; Enhance stomatal conductance; Change of metabolism; Alter of root architecture; Modulation of root exudates; Activate the mechanisms of strengthening cell walls. Decrease rate of transpiration; sensitivity of the plants to water deficiency. Resistance to frost, insect and pathogen attack, disease and pests; enhance locally plant immunity against viruses; reduced virus infection; reduction in root-knot nematode infestation; against salinity stress; water stress; induce improvement of plant growth under sea water stress. Tolerant to iron deficiency.	Increase number of fruits per plant and size of fruit; fruit and crop yield; fruit quality; development of a vigorous root system and improved growth; increase in fresh weight, grain weight and yield components; root formation; growth characters (length, fresh, dry weight) of shoots and roots; quality of the plants; stimulate the growth; induce rooting.
**References**: Aitken and Senn, 1965; Featonby-Smith and van Staden, 1983; Painter, 1983; Finnie and van Staden, 1985; Nelson and van Staden, 1985; Sanderson and Jameson, 1986; Hirsch et al., 1989; Crouch et al., 1992; Verkleij, 1992; Crouch and van Staden, 1993a,b; Nabil and Cosson, 1996; Stirk and Van Staden, 1996, 2006; Herbreteau et al., 1997; Wu et al., 1998; Zhang and Schmidt, 2000; Mercier et al., 2001; Stirk et al., 2003, 2014; Vernieri et al., 2005; Abd El-Baky et al., 2008; Leal et al., 2008; Rayorath et al., 2008; Khan et al., 2009, 2011; Rathore et al., 2009; Craigie, 2011; Vera et al., 2011; Zodape et al., 2011; Sharma et al., 2012a,b, 2014; Abbas, 2013; Arthur et al., 2013; Chbani et al., 2013; Ghannam et al., 2013; González et al., 2013, 2014; Janas and Posmyk, 2013; Jannin et al., 2013; Thomas et al., 2013; Brice-o-Domínguez et al., 2014; Hernandez-Herrera et al., 2014; Michalak and Chojnacka, 2014; Mikiciuk and Dobromilska, 2014; Petrozza et al., 2014; Stadnik and de Freitas, 2014; Vijayanand et al., 2014; Vinoth et al., 2014; Aremu et al., 2015b; Omar et al., 2015; Rengasamy et al., 2015a,b; Satish et al., 2015.					
**4. HIGHER PLANTS**					
*Agapanthus africanus, Allium sativum, Brassica juncea, Brassicacea, Camellia sinensis, Castanea sativa, Ceratonia siliqua, Digitalis sp., Fabaceae, Helianthus annuus, Lupinus albus, Lupinus* sp., *Lycopersicon esculentum, Lycopersicon sp., Malus sp., Medicago sativa, Moringa oleifera, Musa acuminata, Nicotiana tabacum, Quercus sessiliflora, Saccharum officinarum, Vaccinium sp., Vitis vinifera, Zea mays*.	Alkaline hydrolysis; aqueous extraction; controlled fermentation; conventional solid−liquid extraction; cool extraction; fully controlled enzymatic hydrolysis; ethanol extraction; microwave extraction; pressurized solvent extraction; solid−liquid dynamic extraction.	^13^C NMR; ^1^H NMR; Bioassays; C, N elemental analysis; column chromatography fractionation; COSY; DEPT; ELISA; FT-IR; GC-MS; HPLC; HMBC; HMQC; HPLC-DAD-MS; HS-SBSE-GC-MS; ICP-OES; liquid-solid extraction; mass spectrometry; NOESY; preparative thin layer chromatography; qualitative thin layer chromatography; Raman spectroscopy; spectra analysis.	Amino acids: alanine; arginine; aspartic acid; cysteine; glutamic acid; glycine; histidine; isoleucine; leucine; lysine; methionine; phenylalanine; proline; serine; thereonine; tryptophan; tyrosine; valine; etc. Auxins: inodole-3-acetic acid (IAA), Indolbutyric acid (IBA), Naphtoxy acetic acid (NAA); carbohydrates: galactose, glucose, mannose, xylose, arabinose, cellulose. catalase; cytokinins: isopentenyladenosine (IPA), kinetin, etc. elements (N, P, K, Na, S, K, Ca, Mg, P, B, Fe, Zn, Cu, Mn, Ni, Cl, Mo, Co, etc.) ellagitannins: Castalagin; Vescalagin; Roburin E; Grandinin + roburin D; Roburin A + B; Roburin C Enzymatic antioxidants; flavanols (catechin, epicatechin), flavonoid compound: 3-[{O-β-D-glucopyranosyl-(1″-3′)-α-L-rhamnosyl-(1″-2′)}-β-D-glucopyranosyloxy]agapanthegenin. flavanone, naringenin (5,7,4′-trihydroxyflavanone), 5,7,3′4′-tetra-O-acetylflavanone, trans-4,2′,4′-Tri-O-acetylchalcone – Isoliqiuritigenin folic acid, free enzymatic proteins furostanol glycosides gibberellins: gibberellic acid; Gibberellin A4 + A7; glycosides, humic acids inositol lignin and hemicellulose moieties, Lipids, Low molecular weight polyphenols: vanillin; syringaldehyde; coniferaldehyde; sinapaldehyde; vanillic acid; syringic acid; gallic acid; ellagic acid. Melatonin nucleosides: purine, pyrimidine, nucleotides, oligosaccharides organic acids organic nitrogen and organic carbon peptides, Peroxidase, phenolic acids: trans-caffeic acid; trans-p-coumaric acid; ferulic acid; trans-caftaric acid; trans-p-coutaric acid; ellagic acid; gallic acid; protocatechuic acid, syringic acid, vanillic acid), stilbenes (piceid, trans-resveratrol), pyrogallol, sinapaldehyde. Polyphenols polysaccharides, protein, saponins; sugars, superoxide dismutase; tannins triacontanol (TRIA), triglyceride vitamins (A, B_1_, B_2_, B_3_, B6 and PP, C, E) Volatile composition: acids (hexanoic, octanoic, decanoic), alcohols (1-hexanol, 3-hexen-1-ol, 1-octen-3-ol, 1-nonanol, linalool, α –terpineol, guaiacol, benzyl alcohol, 2-phenylethanol), aldehydes (benzaldehyde, nonanal, vanillin), furanic compounds (furfural, 5-hydroxymethylfurfural, 5-methylfurfural, 2-furanmethanol, methyl furoate), lactones (trans-whiskey lactone, cis-whiskey lactone), D –limonene, geranyl acetone, linalyl acetate, β –ionone, stilbene1,2. Volatile compounds: 5-hidroxymethylfurfural; 6-methoxyeugenol; acetovanillone; benzaldehyde; cis-β-methyl-γ-octalactone; eugenol; furfural; guaiacol; trans-β-methyl-γ-octalactone.	Increase of nitrogen assimilation. Increase phosphate uptake. Induction of morphological changes in root architecture. Decrease in accumulation of Na+ and Cl- into shoots under moderate saline conditions. Auxin-, cytokinin-, gibberellin-like activity. Regulation of hormonal system. Regulation of gene expression. Improve photosynthetic rate. Increasing the efficiency of light utilization and dissipation of excitation energy in the PSII antennae. Increase chlorophyll and carotenoids content. Stimulate plant nitrogen and carbon metabolisms. Increase of functional activity of nucleoli of meristematic cells. Increase biochemical contents; leaf nitrogen content; protein amount; free amino acids; carbohydrates; the total sugars; contents of lectin; NADP+; an increase in phenolics in plant tissues; ascorbic acid, β-carotene, elements (N, P, K, Ca, Mg, Fe, etc.). Higher concentrations of macronutrients in the plant tissue. Increase in osmolytes. Changes in sterols, terpenes, glucosinolates composition. Increase SPAD index. Regulation of enzyme activity. Modulating enzymatic and non-enzymatic antioxidant systems. Effects on phenylpropanoid metabolism. Activation of antioxidant defense system. Improved water use efficiency. Regulation of stomata. Enhance plant resistance to stress conditions. Significant antimicrobial activity, especially antifungal activity. Reduce numbers of root knot nematodes. Enhance plant resistance to nematodes. Alleviate the effect of drought; salinity; ameliorate salinity-induced adverse effects.	Growth stimulating effect. Promoting plant growth, health or yield. Increase seed germination; in coleoptile elongation rate; plant biomass, the shoot, root dry weight, root length, and root area, the total dry biomass, plant growth. Ability to manipulate early seedling growth. Rooting. Plant height, number of flowers and number of fruits per plant. Maturity. Enhance the yield of vegetable crops. Strong positive effects on growth, development and fruit quality. Increase in the organoleptic and quality food parameters.
**References:** Murch and Saxena, 2002; Ciesiołka et al., 2005; Fleming et al., 2006; Parrado et al., 2007, 2008; Pretorius, 2007, 2013; Viriji, 2007; Schiavon et al., 2008; Ertani et al., 2009, 2011b, 2013b, 2014; Vyas et al., 2009; Apone et al., 2010; Rivera et al., 2010; Parađiković et al., 2011; Van der Watt and Pretorius, 2011; Yakhin et al., 2011a,b, 2012; De Lucia and Vecchietti, 2012; Hanafy et al., 2012; Abdalla, 2013; Bargiacchi et al., 2013; Christofoletti et al., 2013; Daniels, 2013; Janas and Posmyk, 2013; Parađiković et al., 2013; Pretorius, 2013; Yasmeen et al., 2013, 2014; Ziosi et al., 2013; Arnao and Hernández-Ruiz, 2014; Baglieri et al., 2014; Caulet et al., 2014; Chambers, 2014; Colla et al., 2014; Lachhab et al., 2014; Sánchez-Gómez et al., 2014; Lucini et al., 2015; Pardo-García et al., 2014; Ugolini et al., 2015.					
**5. ANIMAL RAW MATERIALS**					
Animal epithelium, by-products deriving from leather manufacture, chicken feathers, chitin-containing waste materials from the seafood industry, epithelial tissue, hemoglobin hydrolysate, hydrolysis of chrome-tanned waste, leather waste by enzymatic hydrolysis, meat flour, secondary processing of leather waste materials - complex process of collagen protein hydrolysis gained from tannery wastes, waste bovine hooves and horns.	Acid hydrolysis; chemical hydrolytic processes; controlled hydrolysis; Enzymatic hydrolysis; thermal hydrolytic processes.	Amino acid analysis; Bioassays; ecotoxicological tests; Fourier transform infrared spectroscopy; gas chromatography coupled with mass spectrometry(GC/MS); sodium dodecyl sulfate polyacrylamide gel electrophoresis.	elements (Na, S, K, Ca, Mg, P, Fe, Zn, Cu, Mn, Ni, B, etc.); fat; free amino acids (aspartic acid, hydroxyproline, threonine, serine, glutamic acid, proline, glycine, alanine, valyne, methionine, isoleucine, leucine, tyrosine, phenylalanine, γ-aminobutyric acid, histidine, ornitine, lysine, arginine, cysteine, cystine, etc.); melatonin; organic matter; peptides; protein; short-chain peptide bound amino acids.	Improve the utilization of nutrients in plants. Induce morphological changes in root architecture. Auxin-, cytokinin-, gibberellin-like activity. Change hormone levels. Affect on biochemical systems that regulate the biosynthesis of natural plant growth regulators. Synergistic effect with exogenous PGR. Induction of gene expression. Increase of enzyme activities. Accelerate major metabolic reactions. Photosynthetic rate. Increase pigment content, proteins, vitamin C, phenolic contents. Enhance contents of potassium, sodium, copper, zinc and iron in vegetal tissues; alter stomatal conductance; CO_2_ assimilation; reduce transpiration. Increase enzymatic activities and soil biodiversity. Anti-stress effect under drought, high temperatures and freezing, mechanical and chemical stress, viral infection. Stimulate the growth and activity of beneficial microbes. Improve antioxidant activity.	Better root growth and development, effects on foliar growth. Increase root and leaf growth. Root formation. Induction of flowering. Improve good fruit setting and reduce fruit drop. Make more uniform fruit weight and size. High yields.
**References:** Miller et al., 1955; Weissabach et al., 1959; Mladenova, 1978; Betti et al., 1992; Mladenova et al., 1998; Murch and Saxena, 2002; Khan et al., 2003; Apone et al., 2006; Maini, 2006; Kauffman et al., 2007; Cambri et al., 2008; Parrado et al., 2008; Ertani et al., 2009, 2013a; Veselá and Friedrich, 2009; Lisiecka et al., 2011; Parađiković et al., 2011; De Lucia and Vecchietti, 2012; Kolomazník et al., 2012; Pecha et al., 2012; Janas and Posmyk, 2013; Migliore et al., 2013; Parađiković et al., 2013; Sharp, 2013; Vaskova et al., 2013; Arnao and Hernández-Ruiz, 2014; Corte et al., 2014; Lachhab et al., 2014; Rodríguez-Morgado et al., 2014; Tejada et al., 2014.					
**6. HUMATE-CONTAINING RAW MATERIALS**					
Compost, humic-like substances extracted from agro-industrial wastes, leonardite, lignin, peat, soil, vermicompost, volcanic soil, waste materials.	Extraction; thermochemolysis.	^13^C NMR; ^1^H NMR; atmospheric pressure chemical ionization-mass spectrometry (APCI-MS); bioassays; CP/MAS; cross-polarization magic angle spinning (CPMAS)-^13^C-NMR; diffuse-reflectance infrared Fourier transform spectroscopy (DRIFT); electronic microscopy; elemental analysis; FTIR; HPLC/MS/MS; HPSEC; pyrolysis-gas chromatography-mass spectrometry; UV–vis.	amino acids; cellulose and hemicelluloses and saccharides; elements: C, H, N, O; Ca, Cu, Fe, K, Na, P, S, Si, Zn, etc. fatty acids; flavonoids; high-molecular humic substances; humate potassium and potassium oxide; humic acid/fulvic acid; humic substances; lignins; lipids; microorganisms; peptides; phenolic acids (protocatechuic acid, p-hydroxybenzoic acid, p-coumaric acid, ferulic acid); phenols; plant hormones: auxin (IAA); Brassinosteroids (Brassinolide, Castasterone, Teasterone, Typhasterol, 28-Homocastasterone, Cathasterone); Cytokinins (tZ, tZR, tZRMP, tZOG, tZROG, cZ, cZR, cZRMP, cZOG, cZROG, DHZRMP, DHZROG, iP, iPR, iPRMP); gibberellins (GA_1_, GA_2_, GA_3_, GA_4_, GA_5_, GA_6_, GA_7_, GA_8_, GA_9_, GA_13_, GA_15_, GA_19_, GA_20_, GA_24_, GA_29_, GA_34_, GA_44_, GA_51_, GA_53_); proteins.	Induce NO (Nitrous Oxide) synthesis. Increase nitrate uptake. Enhance nutrient uptake and nutritional status. Increase root-to-shoot translocation of elements. Auxin-, cytokinin-, gibberellin-like activity. Regulate of hormonal status. Regulate of gene expression. Ability stimulate various metabolic pathways. Changes in primary and secondary metabolism. Increased chlorophyll a, b and total carotene content. Regulation of photosynthesis, carbon (C) metabolism. Increase assimilation of N, C, and S. Increase protein; phenol content, polyamines. Stimulate the activity of enzymes. Enhance phenylpropanoid metabolism. Alter REDOX homeostasis. Enhance water, salinity and heavy metal stress tolerance. Changes on root architecture. Stimulate of chloroplast division. Alter microorganism communities in the rhizosphere.	The activation of growing processes. Increase root and leaf growth. Increase growth characters (length, fresh, dry weight) of shoots and roots. Increase root size, branching. General increase of biomass. Increase total and marketable yields.
**Reference:** Cacco and Dell'Agnola, 1984; Sanders et al., 1990; Russo and Berlyn, 1991; Adani et al., 1998; Kelting et al., 1998; Zhang and Schmidt, 2000; Canellas et al., 2002, 2010; Chen et al., 2004; Chambolle, 2005; Nardi et al., 2005, 2006, 2007; Zandonadi et al., 2007, 2010; Aguirre et al., 2009; Vasconcelos et al., 2009; Dobbss et al., 2010; Mora et al., 2010; Schiavon et al., 2010; Trevisan et al., 2010; Cordeiro et al., 2011; Ertani et al., 2011a, 2013c; Aydin et al., 2012; García et al., 2012, 2014; Jannin et al., 2012; Abbas, 2013; Pizzeghello et al., 2013; Berbara and García, 2014; Billard et al., 2014; Canellas and Olivares, 2014; Aremu et al., 2015a; Hernandez et al., 2015.					

## Legislation and legal framework

Registration of products used in agriculture is crucial to ensure their practical, safe and legitimate application. In the absence of a sound definition of biostimulants as a discrete group of products (Basak, 2008), the registration procedure and subsequent classification regime is untenable and this inevitably creates a barrier to trade and development. Various countries, states, and administrative regions have developed different categories for registration of potential biostimulants including terminology such as plant conditioners, “other fertilizers,” supplements, soil improvers, plant strengtheners, fitofortificants, etc. (Basak, 2008; Torre et al., 2013; Traon et al., 2014). In many jurisdictions regulatory practices require an itemized description and identification of substances in all commercial product classifications while in others the registration of non-fully identified substances is allowed if those products are considered of complex composition. There is even a proposal for complex biostimulants to not specify the chemical name (IUPAC) and note as “None” with the definition that “this product is a complex mixture of chemical substances” (Traon et al., 2014). If we accept the concept that a biostimulant is a product of clear benefit but unknown mode of action, then it can only be regulated by its safety and proof of efficacy. For example, in pharmacology it has been suggested that “the demand to demonstrate the mode of action of each single component in a phytopharmaceutical may not be obligatory any more” (Ulrich-Merzenich et al., 2009).

**Figure 1:**
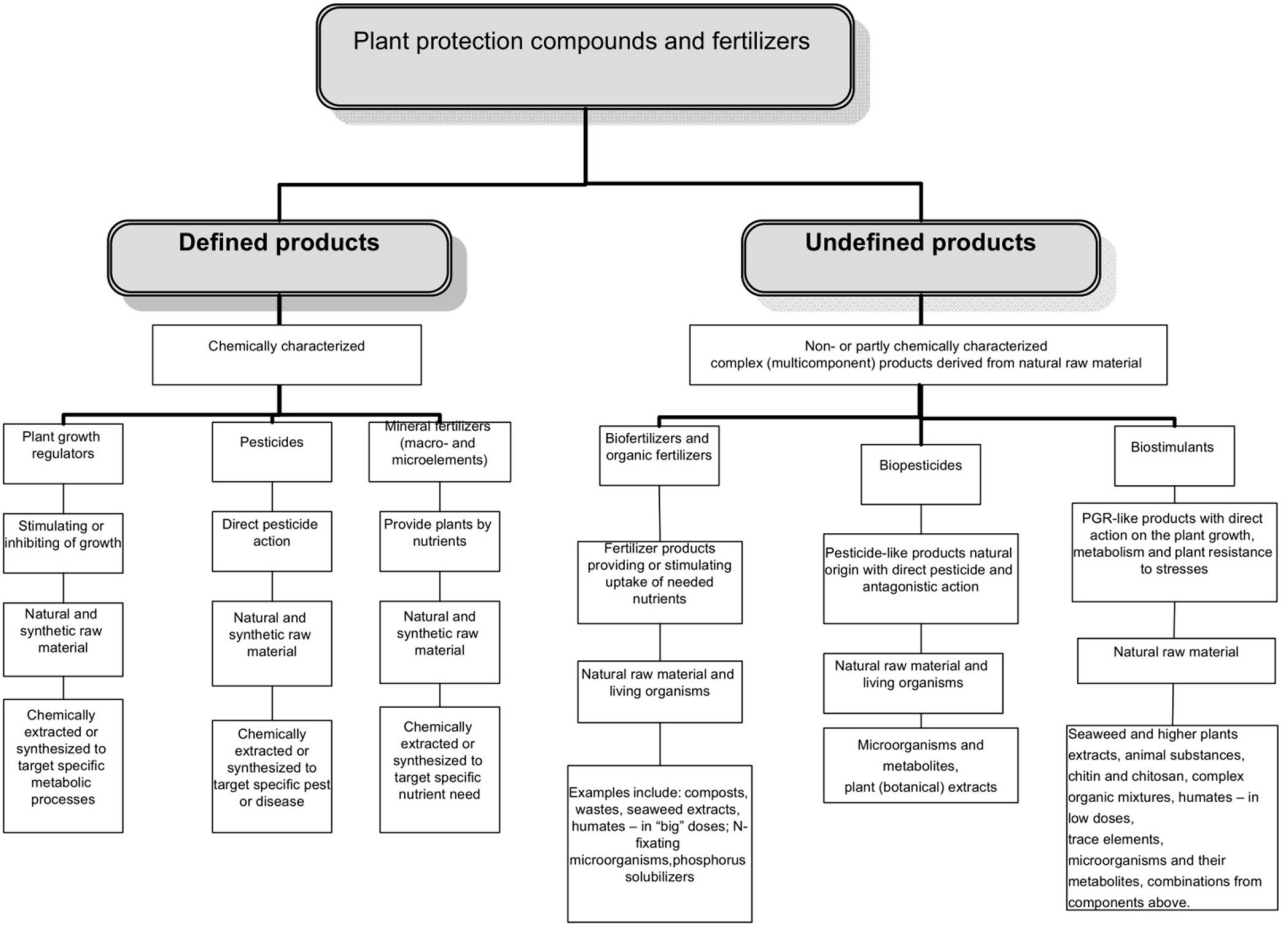
**The distribution of various categories of products among the plant protection products and fertilizers**.

The complex multicomponent nature of many biostimulants clearly complicates discovery of their modes/mechanisms of action, production, registration and use. What is clearly needed however, is a regulatory mechanism to ensure that the products are “generally recognized as safe,” have “a positive benefit on crop productivity” and are discrete from exisiting categories of products. The task of identifying function and agronomic utility can then be pursued independently and will be driven by the marketplace imperative for product quality and consistency. Coordinating national legislation within this framework will become critical for the optimization of biostimulants and trade between different countries. The possible place of biostimulants in the regulatory system of pesticides and agrochemicals is illustrated in Figure 1.

## Primary sources of raw materials

We have conducted an exhaustive analysis of the literature and categorized the majority of the reported biostimulants by origin (Table 4). Microorganisms are widely used for the production of biostimulants and may be derived from bacteria, yeasts, and fungi. These preparations may include living and/or non-living microorganisms and their metabolites. The concept of microorganism-based preparations as biostimulants is described by Xavier and Boyetchko (2002), Sofo et al. (2014), Colla et al. (2015), Matyjaszczyk (2015), and Ravensberg (2015). Different species of algae, mostly seaweeds, are also commonly used for producing biostimulants. Seaweed-based preparations as biostimulants are described in reviews by Crouch and van Staden (1993a), Khan et al. (2009), Craigie (2011), Sharma et al. (2014); and experimental papers by Goatley and Schmidt (1991), Jannin et al. (2013), Billard et al. (2014), Aremu et al. (2015b). Raw materials for biostimulants are also commonly based on higher plant parts including seeds, leaves, and roots and exudates from families *Amaryllidaceae, Brassicacae, Ericaceae, Fabaceae, Fagaceae, Moringaceae, Plantaginaceae, Poaceae, Rosaceae, Solanaceae, Theaceae, Vitaceae*, among others (Naumov et al., 1993; Yakhin et al., 1998, 2011a, 2012, 2014; Pretorius, 2007, 2013; Parrado et al., 2008; Apone et al., 2010; Ertani et al., 2011a, 2013a, 2014; Colla et al., 2014; Yasmeen et al., 2014; Lucini et al., 2015; Ugolini et al., 2015). Biostimulants may also be based on protein hydrolysates and amino acids of animal origin including wastes and by-products (Mladenova et al., 1998; Maini, 2006; Kolomazník et al., 2012; Ertani et al., 2013b; Rodríguez-Morgado et al., 2014), and insect derived chitin and chitosan derivatives (Sharp, 2013). Humate-based raw materials are widely used to derive biostimulants and have been reviewed by Sanders et al. (1990), Kelting et al. (1998), Ertani et al. (2011b), and Jannin et al. (2012). A final category of biostimulants includes those derived from extracts of food waste or industrial waste streams, composts and compost extracts, manures, vermicompost, aquaculture residues and waste streams, and sewage treatments among others. Because of the diversity of source materials and extraction technologies, the mode of action of these products is not easily determined.

## Technologies of production

The technologies used in the production and preparation of biostimulants are highly diverse and include cultivation, extraction, fermentation, processing and purification, hydrolysis, and high-pressure cell rupture treatment (Table 4). In some instances, a biostimulant product may also contain mixes of components derived from different sources and production methods. Frequently the rationale for utilizing extracts rather than raw biomass is a consequence of the need for a standardized manufacturing process to produce a uniform commercial product (Michalak and Chojnacka, 2014). For many products, the production processes are driven by process and marketing demands and are not the result of a targeted strategy to optimize the biological efficacy of the commercial product. While the ultimate composition and possible function of commercial biostimulant products may be partially determined by both the source of raw material and the process by which it is prepared (Traon et al., 2014), there may be manufacturing processes and product treatments utilized that result in compounds that are not present in the initial (primary raw) material. An example of this is the multitude of commercial seaweed extracts, often derived from the same species, that are rarely equivalent (Craigie, 2011). Commercial biostimulant manufactured from similar sources are usually marketed as equivalent products, but may differ considerably in composition and thereby in efficiency (Lötze and Hoffman, 2016). Many manufacturers do not reveal the technology of biostimulant production since that is a commercial secret (Traon et al., 2014).

## Bioactive components and methods of quality control

A diversity of substances contained in raw materials is used for the production of biostimulants. Whereas, primary metabolites are contained in most preparations *de facto*, the presence of secondary metabolites is more specific and depends to a large extent on the raw material used (species, tissue, growing conditions). Primary metabolites include amino acids, sugars, nucleotides, and lipids (Aharoni and Galili, 2011). Secondary metabolites are formed from different primary metabolic pathways, including glycolysis, the tricarboxylic acid cycle (TCA), aliphatic amino acids (AA), the pentose-phosphate and shikimate pathways which are primarily the source of aromatic AA and phenolic compounds (PC), terpenoids/isoprenoids, nitrogen-containing compounds (alkaloids), sulfur-containing compounds (glucosinolates); (Aharoni and Galili, 2011). Frequently, biostimulants are shown to have a multicomponent composition and may include plant hormones or hormone-like substances, amino acids, betaines, peptides, proteins, sugars (carbohydrates, oligo-, and polysaccharides), aminopolysaccharides, lipids, vitamins, nucleotides or nucleosides, humic substances, beneficial elements, phenolic compounds, furostanol glycosides, sterols, etc. (Table 4). While many articles have attempted to describe the composition of complex biostimulants, these descriptions are frequently incomplete since the vast majority of biological molecules that would be present in crude extracts of complex origin, have not yet been characterized and the mere presence of a specific compound does not *a priori* demonstrate that compound is functional. The composition of most biologically derived biostimulant feed stock will also vary with the season of production, species, physiological state of the source organism and growth conditions. Indeed, there is an implication in the marketing of many biostimulants that stress conditions experienced by the plant or microbe utilized to produce the biostimulant, results in the production of stress metabolites and amino acids with consequent beneficial effects on plant response. In the absence of knowledge of the functional component of a biostimulant, changes in composition of a biostimulant over time and between batches and commercial sources cannot be interpreted. In the most rigorously prepared biostimulants from leading companies, high-throughput analytical methods have been employed to ensure consistent product quality (Sharma et al., 2012b). Methods such as chromatography, mass spectrometry, NMR spectroscopy, elemental analysis, ELISA, spectrophotometry, etc. are typically used for this purpose (Table 4). The complexity of this challenge is illustrated in the analysis of a four-year algae composition sequence using a profile or fingerprint technique employing NMR (Craigie et al., 2009).

## Function and effects on whole plants

Biostimulants have been used at all stages of agricultural production including as seed treatments, as foliar sprays during growth and on harvested products. The mode/mechanisms action of “biostimulants” is equally diverse and may include the activation of nitrogen metabolism or phosphorus release from soils, generic stimulation of soil microbial activity or stimulation of root growth and enhanced plant establishment. Various biostimulants have been reported to stimulate plant growth by increasing plant metabolism, stimulating germination, enhancing photosynthesis, and increasing the absorption of nutrients from the soil thereby increasing plant productivity (Table 4). Biostimulants may also mitigate the negative effects of abiotic stress factors on plants and marked effects of biostimulants on the control of drought, heat, salinity, chilling, frost, oxidative, mechanical, and chemical stress, have been observed (Table 4). Alleviation of abiotic stress is perhaps the most frequently cited benefit of biostimulant formulations. The following text describes the primary modes/mechanisms of action that have been demonstrated or claimed for biostimulants in the primary scientific literature.

## Modes of action/mechanisms of action

Understanding the modes of action of an agricultural chemical has been a fundamental requirement for effective marketing and frequently a regulatory requirement for manufactured products used in agriculture. Mode of action is used here to mean “a specific effect on a discrete biochemical or regulatory process,” thus the “mode of action” of Glyphosate is to inhibit the activity of the enzyme enolpyruvylshikimate-3-phosphate synthase (EPSPS). Biostimulants frequently do not meet this standard of specificity and indeed there are few biostimulant products for which a specific biochemical target site and known mode of action has been identified. For a small subset of biostimulants, however, a demonstrated impact on general biochemical or molecular pathways or physiological processes, termed here as a “mechanism of action,” has been identified even though the explicit “mode of action” may not be known. An example of a “mechanism of action” would be a stimulation of photosynthesis or the down regulation of a plant stress signaling pathway without an understanding of the explicit biochemical or molecular “mode of action.”

For many biostimulant products, however, neither a specified mode of action, nor a known mechanism of action, has been identified. The presence of some spurious products in the marketplace compromises the market for all players resulting in the assumption by many, that biostimulants as a whole, are “snake oils” (Basak, 2008), a pejorative term implying the product is of no value. Multicomponent biostimulants are particularly difficult to reconcile since they may have constituents for which the mode of action is known and components of no known functional benefit. Furthermore, multicomponent biostimulants will frequently contain measureable but biologically irrelevant concentrations of known essential elements, amino acids, and plant hormones etc., for which the mode of action is known but the concentrations are irrelevant when used at recommended rates. Thus, for many of the multicomponent biostimulant in the marketplace today, we propose that a demonstration of a clear “mechanism of action” is a more rationale and attainable regulatory goal than requiring an unequivocal demonstration of the “mode of action.”

Insight into the use of the terms “mode and mechanism” of action can be drawn from the pesticide science and pesticide development. In pesticide science, the “mechanism of action” describes the integral of all the biochemical events following application while the “mode of action” characterizes the main features of a bioactive molecule and its specific biochemical action leading to its effect in treated plants (Aliferis and Jabaji, 2011). In reference to plant bioregulators, Halmann (1990) suggests that ideally an understanding of the mode of action of plant bioregulators on the molecular level requires the identification of the receptor site for each regulator, as well as the elucidation of the subsequent reactions. In reality this standard is often not met in biopesticide or biostimulant products where the identification of the molecular targets of all bioavailable (and frequently uncharacterized) compounds within a given extract cannot be easily achieved. The identification of the target binding sites of the natural biomolecules has, however, proven to be helpful in the design of new insecticidal molecules with novel modes of action (Rattan, 2010).

At the present time, given the difficulty in determining a “mode of action” for a complex multicomponent product such as a biostimulant, and recognizing the need for the market in biostimulants to attain legitimacy, we suggest that the focus of biostimulant research and validation should be upon determining the mechanism of action, without a requirement for the determination of a mode of action. This is the standard of practice for many pharmacological products. With the development of advanced analytical equipment, bioinformatics, systems biology and other fundamentally new methodologies a more complete understanding of the mechanisms and even possible modes of action of these materials may be achieved in the future. While this proposal suggests that the development and marketing of a biostimulant may not require a demonstration of the mode of action, it is still in the interest of the manufacturers of these products to pursue an understanding of the mode of action so that the product can be improved and the use can be optimized for various environments and cropping systems.

The mechanisms of action of all but a few biostimulants remain largely unknown (Rayorath et al., 2008; Khan et al., 2009; Rathore et al., 2009). This is primarily due to the heterogeneous nature of raw materials used for production and the complex mixtures of components contained in biostimulant products which makes it almost impossible to identify exactly the component(s) responsible for biological activity and to determine the involved mode(s) of action (Parađiković et al., 2011). Therefore, focus should be upon the identification of the “mechanisms of action” of biostimulants as indicated by general positive impacts on plant productivity through enhancement in processes such as photosynthesis, senescence, modulation of phytohormones, uptake of nutrients and water, and activation of genes responsible for resistance to abiotic stresses and altered plant architecture and phenology (Khan et al., 2009; Sharma et al., 2012b). An example of this process is the advances in use of protein-based biostimulants for which recent studies have identified the target metabolic pathways and some of the mechanisms through which they exert their effects on plants (Nardi et al., 2016).

To further our understanding of modes/mechanisms of biostimulant action we have systematized the stages of biostimulants action on plants after their application: (1) penetration into tissues, translocation and transformation in plants, (2) gene expression, plant signaling and the regulation of hormonal status, (3) metabolic processes and integrated whole plant effects.

## Penetration into tissues, translocation, transformation in plants

The penetration of amino acids and peptide based biostimulants into plant tissues has been investigated using radiolabeled amino acids (Maini, 2006) and mathematical modeling (Kolomazník et al., 2012; Pecha et al., 2012). The components of a biostimulant preparation of animal origin, labeled with ^14^C proline and glycine, were shown to penetrate rapidly into treated leaves and where subsequently distributed to other leaves (Maini, 2006). The mathematical model based on the “mechanism of diffusion” allows the estimation of the time required for the absorption of a minimal amount of the active component of a biostimulant. Furthermore, it describes the process of its transport from the moment of penetration into the leaf until the arrival at more distant tissues (Kolomazník et al., 2012; Pecha et al., 2012). The penetration of protein hydrolysates into a plant tissue occurs via diffusion of protein molecules through membrane pores (Kolomazník et al., 2012) and is energy-dependent (Parrado et al., 2008). Biostimulants must have a good solubility in water or other suitable solvents. This is a precondition for most types of application and for sufficient penetration of active ingredients into internal structures of treated plants. Surfactants and other additives may be required to overcome solubility and uptake limitations including lipophilicity and molecular size of active components (Kolomazník et al., 2012; Pecha et al., 2012).

## Gene expression, signaling, and hormone interactions

Ultimately a full understanding of the biological activity of complex biostimulant preparations will require a detailed understanding of the mechanism of action and effects on plant productivity and the identification of the biologically active molecules and their molecular mode of action (Henda and Bordenave-Juchereau, 2014). A wide array of molecular methods has been used to attempt to discern the active compounds found in biostimulants including microarrays, metabolomics, proteomic, and transcriptomics methods. These technologies have been applied to biostimulants to probe changes in gene expression following the application of biostimulants (Jannin et al., 2012, 2013; Santaniello et al., 2013). Further research on the effects of complex biostimulants and their components on the complete genome/transcriptome of plants will be required to understand the mechanisms of action involved in growth responses and stress mitigation (Khan et al., 2009). The search for the mode of action of biostimulants is complicated by the observation that many biostimulants have been shown to induce genes and benefit productivity only when plants are challenged by abiotic and biotic stress. Experimental methods must therefore be developed to produce relevant and reproducible stress conditions so that the application of any molecular tool to probe gene function produces results that are relevant to the purported effects on plant productivity.

The role of signaling molecules in plant response to environmental cues has been an area of active research in plant biology. The process of signal transmission involves the synthesis of signaling molecules (ligands), their translocation, their binding to receptors, the resulting cellular responses, and, finally, the degradation of the signaling molecules (Zhao et al., 2005; Wang and Irving, 2011). When the signaling molecule binds to its receptor, the initial cellular response is the activation of secondary messengers, or intracellular signaling mediators, which cause a further series of cellular responses. Among the substances that may act as secondary messengers are: lipids, sugars, ions, nucleotides, gases, Ca^2+^, cAMP, cGMP, cyclic ADP-ribose, small GTPase, 1,2-diacylglycerol, inositol-1,4,5-triphosphate, nitric oxide, phosphoinosides, and others (Zhao et al., 2005; Wang and Irving, 2011). Generally, a membrane-mediated action is typical for water-soluble compounds, while cytosol-mediated activity is primarily triggered by lipophilic compounds.

Whereas, enzymes interact with their substrates in a geometrical way (“lock and key”), signaling molecules are thought to have a topochemical affinity to their receptors. It is assumed that the interaction of such components at the receptor site is cooperative and quantized (Gafurov and Zefirov, 2007). The bioactive compounds in some biostimulants are also proposed to display signaling activity in plants or induce signaling pathways. Various amino acids (Forde and Lea, 2007; Arbona et al., 2013), and peptides (Ivanov, 2010) function as signaling molecules in the regulation of plant growth and development (Ertani et al., 2009; Mochida and Shinozaki, 2011). Peptide signaling is important in various aspects of plant development and growth regulation including meristem organization, leaf morphogenesis, and defense responses to biotic and abiotic stress (Schiavon et al., 2008). Specific signaling peptides contained in a plant-derived protein hydrolysate have been shown to affect plant growth and development, defense responses, callus growth, meristem organization, root growth, leaf-shape regulation, and nodule development (Matsubayashi and Sakagami, 2006; Colla et al., 2013). Protein hydrolysates from soybean and casein have been shown to act as elicitors to enhance grapevine immunity against *Plasmopara viticola* (Lachhab et al., 2014).

Proteins may also contain hidden peptide sites, “cryptides” or “crypteins” in their amino acid sequence, which may have their own biological activities, distinct from its precursor (Ivanov, 2010; Samir and Link, 2011). Evidence that cryptides can trigger plants defense reactions have recently been demonstrated (Yamaguchi and Huffaker, 2011) and there are reports of the isolation of cryptides by hydrolysis of proteins from marine organisms, including seaweeds, and cryptides may be present naturally in a variety of biological derived products (Henda and Bordenave-Juchereau, 2014; Hayes et al., 2015).

Many small molecular weight substances are known to participate in signaling cascades *in vivo*. Exogenous amino acids may affect biological processes by acting directly as signal molecules or by influencing hormone action via amino acid conjugation (Tegeder, 2012). It has been suggested that amino acid based biostimulants are readily absorbed and translocated by plant tissues and once absorbed, they have the capacity to function as compatible osmolytes, transport regulators, signaling molecules, modulators of stomatal opening, and may detoxify heavy metals among other benefits (Kauffman et al., 2007). Sugars (Smeekens, 2000; Eveland and Jackson, 2012) and fatty acids and plant lipids (Kachroo and Kachroo, 2009) are also known to act as signaling molecules and mitigators of stress response in plants (Okazaki and Saito, 2014). Animal based lipid soluble fractions, have also been observed to produce an auxin-like response (Kauffman et al., 2007), while sugars, sucrose, and its cleavage products (hexoses), are also known to act as signaling molecules through regulation of gene expression and by interaction with other hormone signals including auxins. In a sunflower meal hydrolyzate, amino acids, humic substances, microelements, and sugars present in the biostimulant appeared to coordinate, with auxin-like compounds in complex signaling cross-talk promoting plant growth, enhancing plant transplanting success and increasing final crop yield (Ugolini et al., 2015).

Hormones are of central importance for the regulation of metabolic processes and plant development in a complex system of interacting hormones and cofactors, the functions of which are closely intertwined and mutually dependent (Wang and Irving, 2011). Biostimulants developed from humic substances, complex organic materials, seaweeds, antitranspirants, free amino acids (Du Jardin, 2012), and crude extracts of lower (Rathore et al., 2009) and higher plants (Yakhin et al., 2012) have been frequently demonstrated to have an effect on plant hormonal status (Kurepin et al., 2014). While hormone-like compounds may be present in biostimulants, it is also possible that *de novo* synthesis of hormones may be induced by such preparations in treated plants (Jannin et al., 2012) and amino acids, glycosides, polysaccharides and organic acids are contained in many biostimulants and may act as precursors or activators of endogenous plant hormones (Parađiković et al., 2011). Hormones or hormone-like effects could therefore be responsible for the action of natural biostimulants derived from microorganisms, algae, higher plants, animal, and humate based raw material (Table 4).

## Metabolic effects

Information on currently available biostimulants gives some insight into the possible biochemical and molecular genetic effects of biostimulants derived from different natural raw materials (Table 4). Many published reports are available suggesting various biostimulants improve plant productivity through increased assimilation of N, C, and S (Jannin et al., 2012, 2013), improved photosynthesis, improved stress responses, altered senescence, and enhanced ion transport (Gajic, 1989; Khan et al., 2009; Parađiković et al., 2011). Biostimulants are also reported to increase free amino acids, protein, carbohydrates, phenolic compounds, pigment levels, and various enzymes (Table 4). The protective effect of many biostimulants against biotic and abiotic stresses has been associated with a reduction of stress-induced reactive oxygen species, activation of the antioxidant defense system of plants, or increased levels of phenolic compounds (Ertani et al., 2011a, 2013a).

While it is clear that many biologically derived biostimulants contain small molecular weight compounds that are involved in signaling events and may directly influence plant metabolic processes, it remains unclear how an exogenous soil or foliar application of an uncharacterized product can have predictable and beneficial responses in plants. It is well-known, for example, that application of exogenous plant hormones or compounds that disrupt hormone function (PGR's) can have markedly negative effects on plants and that optimization of PGR materials and their applicaitons requires precise information on dosage and timing. Application of biostimulants for which the dosage and efficacy of the functional compounds is unknown, cannot, therefore, be expected to result in predictable plant responses and identification of molecules with effects on plant metabolic processes is not, in of itself, a sufficient explanation for the function of a biostimulant. It is also uncertain why the application of a biostimulant with purported function as a PGR, signaling molecule or other discrete compound would be superior to, or more easily controlled, than a direct application of the purified product itself.

## Toxicological and ecological aspects

Modern crop production requires a balance of high and consistent productivity with maximum safety for consumers, agricultural workers, and the environment (Rathore et al., 2009; Jannin et al., 2012; Pecha et al., 2012). While some biostimulants have been analyzed with regard to unwanted side effects including negative impact on the natural environment (Janas and Posmyk, 2013) most biostimulants have not been fully characterized but have been regarded as generally recognized as safe (GRAS in the US) on the basis of the biological origin of their constituents (Thomas et al., 2013). Generally, biostimulants are assumed to be biodegradable, non-toxic, non-polluting and non-hazardous to various organisms. While this may be a rational conclusion for many formulations derived from biological materials such as seaweed extracts and their components (Turan and Köse, 2004; Dhargalkar and Pereira, 2005; Rathore et al., 2009; Michalak and Chojnacka, 2014; Stadnik and de Freitas, 2014), higher plants (Onatsky et al., 2001; Abdalla, 2013; Yakhin et al., 2013), chitin and chitosan (Bautista-Baños et al., 2006; Cabrera et al., 2013) it is not clear that this is a valid assumption for microbial products or products that would not normally be present in agricultural fields.

Biostimulants have been utilized as bioremediants and have been shown to improve ATP levels and phosphatase and urease activity (Tejada et al., 2011a), and hence increase the rate of degradation of xenobiotics in the soil (Tejada et al., 2010, 2011b) and to enhance beneficial soil microbial communities under semi-arid climates (Tejada et al., 2011b). Biostimulants may also help reduce the amount of potentially risky agrochemicals (Kolomazník et al., 2012) including reducing the use of fertilizers and pesticides (Hamza and Suggars, 2001). Most compounds contained in biostimulants are natural constituents of terrestrial and aquatic ecosystems (Jannin et al., 2012) and metabolites of plant and microbial origin and as such most are generally regarded as safe, particularly at the low rates at which they are typically applied. Thus, it has been proposed that biostimulants can be positioned as eco-friendly products for sustainable agriculture (Mladenova et al., 1998; Ertani et al., 2011a; Ghannam et al., 2013; Vijayanand et al., 2014). In many countries, however, biostimulants are not subject to rigorous toxicological screening (Traon et al., 2014) and there remains the potential for the persistence of human pathogens in materials of animal origin and for the synthesis of novel compounds of unknown function or toxicology during the manufacturing process.

## Economic aspects

Even though there have been relatively few rigorous demonstrations of the benefit of biostimulants, and to a large extent the mode of action of these products remains uncertain, the industry for biostimulants is substantial and rapidly growing. Though many recent “market” studies show that the market for these products is growing at a remarkable rate, the validity of these analyses must be considered with care as they frequently do not provide an explicit definition of term “biostimulants.” The value of the European biostimulants market ranged from €200 to €400 million in 2011, €500 million in 2013 and may grow to more than €800 million in 2018 with annual growth potential in 10% and more (EBIC, 2011a, 2013; Traon et al., 2014). France, Italy, Spain are the leading EU countries in the production of biostimulants (Traon et al., 2014). In North America, the biostimulant market was valued at $0.27 billion in 2013^1^, and is expected to grow at a growth rate of 12.4% annually, to reach $0.69 billion by 2018, the USA is the largest producer and consumer of biostimulants in the region (http://www.micromarketmonitor.com/). In 2014, the USA market was assessed at $313.0 million and is projected to reach $605.1 million by 2019^2^, at a CAGR of 14.1% (http://news.agropages.com/). The biostimulants market in the Asia-Pacific was valued at $0.25 billion in 2013, and is expected to grow at a CAGR of 12.9% annually, to reach $0.47 billion by 2018 (Asia Biostimulants Market, 2015)^3^. China and India are key countries playing a significant role. The Southeast Asian & Australasian biostimulants market was valued at $233.8 million in 2015, and is projected to reach $451.8 million by 2021 (http://news.agropages.com/)^4^. The market in Latin America was valued at $0.16 billion in 2013^5^, and is expected to grow at a CAGR of 14.4% annually, to reach $0.32 billion by 2018 (http://www.micromarketmonitor.com/). This market is mostly concentrated in Brazil and Argentina. The regional market shares of the global biostimulants market^6^ are: EU—41.7%, North America—21.5%, the Asia-Pacific region—20%, Latin America—12.9%. Globally, it biostimulants were valued at $1402.15 million in 2014 and are projected to have aCAGR of 12.5% reaching $2524.02 million by 2019^7^, largely as a consequence of growing interest in organic products. Wu (2016) summised that “the global biostimulants market is projected to reach $2.91 billion by 2021, with a CAGR (compound annual growth rate) of 10.4% from 2016 to 2021. In terms of area of application, the biostimulants market is projected to reach 24.9 million hectares by 2021 and is projected to grow at a CAGR of 11.7% from 2016 to 2021” (Wu, 2016).”

## Problems and prospects

The biostimulant industry faces many problems and challenges. Until recently biostimulant products based on natural raw materials and particularly waste stream has mainly been developed based on observational and less commonly, empirical data. While many contemporary biostimulants have been shown to be effective in practice, very few biostimulants can claim to understand the mechanisms or modes of action (Khan et al., 2009). Furthermore, while biostimulants can be categorized by source of origin, this is frequently inadequate as very substantial differences can exist between products even within a common feed stock origin. The challenge to biostimulant science is further exacerbated since composition and content of active substances in the original plant raw material can be affected by many factors including the location and growing conditions, season, species, variety, organ, and the phase of growth (Naumov et al., 1993; Dragovoz et al., 2009; Sharma et al., 2012b). Similarly, the response of the target crop can be expected to vary across crops and environments. One solution to this problem is to derive the raw materials for the biostimulant under highly regulated conditions. This approach has been successfully implemented by leading seaweed producers and fermentation based products that have developed harvesting and manufacturing processes that ensure uniformity of product performance through time. The development of a product with uniformity of response is not, however, a guarantee that the product is optimized for biological efficacy.

To address these issues, developments in -omics approaches will be critical in accelerating the discovery of mode of action of bioactive compounds (Aliferis and Jabaji, 2011; Craigie, 2011; Jannin et al., 2012) and optimizing their use. Metabolomics, phenomics and agronomics represent the integration of gene expression, protein interactions, and other regulatory processes as they impact on plant productivity and thus are more appropriate tools for discovery in this field than mRNA, transcripts, or proteins analyzed in isolation (Arbona et al., 2013). Integrative, multidisciplinary approaches using tools from transcriptomics in conjunction with metabolomics and biochemical analysis are necessary to establish the mechanism of action and to identify the active components in the extracts (Lee et al., 2012). The difficulty in identifying modes of action and subsequent standardization of composition of multicomponent biostimulants based on natural raw materials will continue to hamper the use, certification and registration of biostimulants. The solution to this problem will require the collaborative efforts of specialists from different fields: chemists, biologists, plant physiologists, industrial manufacture, sales and distribution and those with expertise in practical agricultural production (Raldugin, 2004; Craigie, 2011; Jannin et al., 2012; Lee et al., 2012).

Products with a single active substance represent a simpler construct in which the physiological effects and mechanism of action can be more readily determined and hence certification and registration is simpler. The multicomponent composition of many preparations, however, are much more difficult to characterize (Bozhkov et al., 1996), though they may offer novel insight into biological synergy (Bulgari et al., 2015), multifunctionality and emergence which may be crucial to product efficacy (Gerhardson, 2002). In the absence of a functional rationale for every constituent in a multicomponent biostimulant, it is likely that there will be molecules present that may positively or negatively influence plant productivity. Currently, it is almost impossible using available chemical-synthetic, and genetic engineering approaches to reproduce the full suite of molecules and complexes of biologically active substances (Kershengolts et al., 2008) that are present in most biostimulants.

## Pro's and con's of biostimulants science and practice

Many have noted the state confusion in the field of biostimulants (Torre et al., 2013; Traon et al., 2014) and this has resulted in the opinion that much of the biostimulant market is not based on science or efficacy and that many products are little more than recycled waste products sold on the basis of pseudoscience and marketing. Indeed, research on several biostimulant products has shown them to be ineffective or to contain inactive, unstable or inconsistent properties with several showing negative effects compared when contrasted with well-designed controls (Csizinszky, 1984, 1986; Albregts et al., 1988; Di Marco and Osti, 2009; Vasconcelos et al., 2009; Banks and Percival, 2012; Cerdan et al., 2013; de Oliveira et al., 2013; Carvalho et al., 2014). For example, foliar and root application of a product containing amino acids from animal origin have been reported to cause severe plant-growth depression and negative effects on Fe nutrition while a second product containing amino acids from plant origin stimulated plant growth (Cerdan et al., 2013). In another report that tested several biostimulant products it was concluded that “none of the biostimulant products tested achieved a sufficient degree of pathogen control to warrant replacement of or supplementation with conventional synthetic fungicides” (Banks and Percival, 2012), and there have been demonstrated positive and negative impacts and overall questions of the economic feasibility of the use of humic substances for increasing crop yields (Rose et al., 2014). Since biological systems are inherently complex, and given that most biostimulant products have not been characterized and have received relatively little replicated and rigorous independent validation, it is perhaps not surprising that many products are ineffective or highly variable in response. Nevertheless, there are a significant number of rigorous independent reports of benefits from some biostimulant formulations and market growth data demonstrates that there is a good deal of support for these products within agricultural producer communities. That such market growth has occured, even in the absence of a known “mechanism of function” suggests that there are aspects of plant metabolism and productivity constraints that are not understood but are potentially important if we are to achieve the goal of increased global food production.

The market euphoria that is taking place in the biostimulant industry recognizes these unknowns and biostimulants are viewed by many innovators and investors as a mechanism to conduct broadscale, if unfocussed, discovery of novel biologically derived molecules. Much as the exploration of marine organisms, and plants and microbes from diverse ecosystems has led to the discovery of novel pharmaceuticals, so too the development of biostimulants from the broad range of source materials, holds significant promise of discovery. Recent years have seen rapid growth in the number of published studies, increased numbers of scientific conferences and development of legal framework and legislation. These trends will inevitably improve the image of this industry and the efficacy of products. Two significant problems still exist within the industry broadly: (1) preparations of products with highly complex multicomponent and incompletely identified composition make the identification of a primary mode of action extremely difficult and (2) the current classification and legislation/legal framework for regulation of biostimulants is based primarily on source material and not on biological mode of action. Hence there is insufficient capacity to differentiate products, and there is the potential for the successful demonstration of a single product within a biostimulant category, to falsely indicate the efficacy of the group as whole.

Several topical questions need consideration in the future:

1. Can living cultures of microorganisms, which have the ability to stimulate the growth of plants be referred to biostimulants?
2. Are non-essential elements that result in improved plant productivity, biostimulants?
3. How should biostimulants with a complex completely unidentified structure where all the components and modes/mechanisms involved have not been established be registered and regulated in national and international legislation?
4. What standard of proof of efficacy is appropriate that both stimulates development and discourages the sale of materials of no benefit?
5. On what principles, should the final classification of biostimulants be based and what categories should it contain?

## Conclusions

Modern biostimulants are complex mixtures derived from raw materials of highly diverse origin utilizing highly diverse manufacturing processes and as such can be expected to have a broad spectrum of possible biological activity and safety. To distinguish biostimulants from the existing legislative product categories including essential nutrients, pesticides, or plant hormones a biostimulant should not solely function by virtue of the presence of elements or compounds of known function. We propose, therefore, a definition of a biostimulant as “a formulated product of biological origin that improves plant productivity as a consequence of the novel or emergent properties of the complex of constituents and not as a sole consequence of the presence of known essential plant nutrients, plant growth regulators, or plant protective compounds.” Consistent with this definition, the ultimate identification of a novel molecule within a biostimulant that is found to be wholly responsible for the biological function of that biostimulant, would necessitate the classification of the biostimulant according to the discovered function.

This novel definition is inspired by three observations: (1) that the development of the biostimulant industry will inevitably result in the discovery of novel biologically active molecules and that the identification and classification of these molecules will benefit biological discovery more greatly if these molecules are explicitly described than if they were merely labeled as “biostimulants,” (2) that there is a need for the nascent biostimulant industry to explicitly discourage the inclusion of nutrient elements and known biologically active molecules under the guise of a “biostimulant” and (3) that there is a need to recognize that classic reductionist biology/chemistry may indeed be insufficient to explain biological complexity (Luisi, 2002; Lüttge, 2012; Bertolli et al., 2014).

The definition provided here is important as it emphasizes the principle that biological function can be modulated through application of complex mixtures of molecules for which an explicit mode of action has not been defined. The definition also requires a demonstration of beneficial impacts of the biostimulant on plant productivity. Given the difficulty in determining a “mode of action” for a biostimulant, and recognizing the need for the market in biostimulants to attain legitimacy, we suggest that the focus of biostimulant research and validation should be upon determining the mechanism of action, without a requirement for the determination of a mode of action. This can be achieved through careful agronomic experimentation, molecular or biochemical demonstration of positive impact on biological processes or the use of advanced analytical equipment to identify functional constituents. Given the prerequisite multi-component and emergent characteristics of biostimulants, the discovery of the mode of action is likely to require application of new techniques in bioinformatics and systems biology. While the definition proposed here suggests that the development and marketing of a biostimulant does not require a demonstration of the mode of action, it is still in the interest of the commercial producers of these products to pursue an understanding of these products so that the product can be improved and optimized for use in various environments and cropping systems.

While there is a clear commercial imperative to rationalize biostimulants as a discrete class of products, there is also a compelling biological case for the science-based development of the biostimulant science that is grounded in the observation that the application of biological materials derived from various organisms, including plants, that have been exposed to stressors can affect metabolic and energetic processes in humans, animals, and plants (Filatov, 1951a,b). This hypothesis is based upon the premise that functional chemical communication occurs between individuals or organs that favorably modulate metabolic pathways and networks at different plant hierarchical levels. Inter and intra organism communication and consequent molecular and metabolic regulation are at the heart of the science of systems biology and the tools of systems biology will inevitably be critical to the realization of mode of action of many biostimulants. Continued investments by commercial entities in biostimulant research and product development will serve as a critical driver of discovery in this realm and will inevitably lead to the identification of novel biological phenomenon, pathways and processes that would not have been discovered if the category of biostimulants did not exist, or was not considered legitimate.

## Author contributions

All authors OY, AL, IY, PB, contributed equally to this review.

### Conflict of interest statement

The authors declare that the research was conducted in the absence of any commercial or financial relationships that could be construed as a potential conflict of interest.

## Footnotes

^1^North America biostimulants market (2015). Available online at http://www.micromarketmonitor.com/ (Accessed February 27, 2015).

^2^North America biostimulants market to reach $605.1 million by 2019 (2015). Available online at: http://news.agropages.com/ (accessed August 18, 2015).

^3^Available online at: http://www.micromarketmonitor.com/ (Accessed February 27, 2015).

^4^Southeast Asian and Australasian Biostimulants Market Trends and Forecasts to 2021 Available online at: http://news.agropages.com/ (Accessed August 15, 2016).

^5^Latin America biostimulants market (2015). Available online at: http://www.micromarketmonitor.com/ (Accessed February 27, 2015).

^6^Global biostimulants product market to reach $2241.0 million by 2018. Available online at: http://news.agropages.com/ (Accessed December 30, 2015).

^7^Global biostimulants market to reach $2.52 bn by 2019. Available online at http://news.agropages.com/ (Accessed December 30, 2015).
